# Lewis basicity generated by localised charge imbalance in noble metal nanoparticle-embedded defective metal–organic frameworks

**DOI:** 10.1038/s41467-018-06828-4

**Published:** 2018-10-18

**Authors:** Ying Chuan Tan, Hua Chun Zeng

**Affiliations:** 1 Department of Chemical and Biomolecular Engineering, Faculty of Engineering, National University of Singapore, 10 Kent Ridge Crescent, Singapore, 119260 Singapore; 2 Cambridge Centre for Advanced Research and Education in Singapore, 1 Create Way, Singapore, 138602 Singapore

## Abstract

Interactions between metal nanoparticles (NPs) and metal–organic frameworks (MOFs) in their composite forms have proven to exhibit beneficial properties, such as enhanced catalytic performance through synergistic effects. Herein, we show that Lewis basic sites can be created within an anionic defective MOF by engineering the electronic state of the pendant carboxylate groups situated at the defect sites. This is achieved from the concerted interactions between the pendant carboxylate groups, embedded Pd NPs and charge-balancing cations (*M*^*n+*^ = Ce^3+^, Co^2+^, Ni^2+^, Cu^2+^, Mg^2+^, Li^+^, Na^+^ or K^+^). This work is the first example of generating a new collective property, i.e. Lewis basicity, in metal-carboxylate MOFs. Importantly, the choice of *M*^*n+*^, used during cation exchange, acts as a convenient parameter to tune the Lewis basicity of the MOF-based nanocomposites. It also provides a facile way to incorporate active metal sites and basic sites within carboxylate-based MOFs to engineer multifunctional nanocatalysts.

## Abstract (web summary)

Interactions between metal nanoparticles (NPs) and metal–organic frameworks (MOFs) in their composite forms have proven to exhibit beneficial properties. Here the authors present a unique approach to immobilise Pd NPs and, more importantly, to generate tunable basic sites within an anionic defective MOF.

## Introduction

Metal–organic frameworks (MOFs), also called porous coordination polymers, belong to a new family of inorganic/organic hybrid porous materials with three-dimensional crystalline structures. Constructed from tunable metal clusters and organic ligands, MOFs can be engineered with highly diverse functionalities for a wide range of applications^1–5^. Heterogeneous catalysis is one of the most explored fields of uses of MOFs^6,7^. For example, MOFs can be used as solid bases, which are crucial in numerous organic reactions^8^. Development of solid bases is industrially important as they represent a greener and safer alternative to liquid bases. To date, nevertheless, MOFs with basic properties are mostly limited to those with N-functionalised linkers that act as Lewis basic sites^9–11^. Development of new strategies to create basic functionality in MOFs could potentially lead to the discovery of more efficient synthetic routes and interesting catalytic properties^12,13^. Other than directly varying metal nodes and organic linkers in the design of MOFs, there must be other methods to tune their functionalities.

MOFs with charged frameworks are an important subclass of the porous materials as they can undergo functionalisation with ease. For instance, being made up of negative-charged scaffolds with replaceable charge-balancing cations grafted within the channels, anionic MOFs can undergo convenient cation exchange. This ion exchange process is an effective method to modify the properties of MOFs for specific applications, such as gas adsorption and separation, catalysis and ionic conduction^14–16^. The integration of metal nanoparticles (NPs) into MOFs is another straightforward approach to introduce additional functionalities^17^. Metal/MOF nanocomposites have gained massive attention as such hybrid catalytic systems could demonstrate synergistic effects and enhanced stability of the NPs^18–26^. On the other hand, there is still no report that investigates the interactions between metal NPs and MOF defect sites. Such defective MOFs have been recognised to exhibit new or enhanced functionalities as compared to pristine MOFs^27,28^. Therefore, there are potentially unexplored properties that could emerge from the interactions between defective MOFs and included metal NPs.

In our previous works, we had developed a synthetic approach that enabled the preparation of MOFs with hierarchical structures^29^. We were also able to obtain ring-like defective HKUST-1 with anionic frameworks and mesoporosity (HKUST-1-R)^30^ that consists of copper nodes coordinated with benzene-1,3,5-tricarboxylate (BTC^3−^) organic linkers and pendant carboxylate groups imprinted with cetyltrimethylammonium ions (CTA^+^) as the charge-balancing species. The formation of pendant, i.e. dangling, carboxylate groups located at the defective sites was due to the development of metal cluster vacancies that arose during the rapid synthesis. Consequently, performing cation exchange between CTA^+^ with various metal ions (*M*^*n*+^) results in *M*-HKUST-1-R with extrinsic mesopores (Supplementary Fig. 1)

**Fig. 1:**
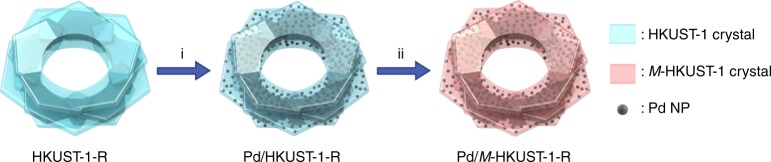
Schematic illustration of the stepwise preparation of Pd/*M*-HKUST-1-R. HKUST-1-R undergoes an in situ reduction of Pd^2+^ to form Pd/HKUST-1-R (step i) and subsequent cation exchange with *M*^*n*+^ (where *M*^*n*+^ = Ce^3+^, Co^2+^, Ni^2+^, Cu^2+^, Mg^2+^, Li^+^, Na^+^ or K^+^) forms Pd/*M*-HKUST-1-R (step ii)

In this work, we present a unique approach to immobilise Pd NPs and, more importantly, to generate tunable basic sites within an anionic defective MOF, thus establishing a new synthetic route to design multifunctional MOF-based nanocomposites. As summarised in Fig. 1, first (step i), HKUST-1-R serves as a host to attract noble metal cations (i.e. Pd^2+^) and to stabilise the resultant NPs after a mild reduction step. This intermediate composite is denoted as Pd/HKUST-1-R. Second (step ii), owing to the anionic property of the MOF support, other metal ions *M*^*n*+^ can be added to carry out cation exchange with the molecular-imprinted CTA^+^ within HKUST-1-R to obtain Pd/*M*-HKUST-1-R. Through careful selection of the charge-balancing cations (*M*^*n*+^ = Ce^3+^, Co^2+^, Ni^2+^, Cu^2+^, Mg^2+^, Li^+^, Na^+^ or K^+^) used during cation exchange, the basicity of MOF composite can be tuned. The creation of basicity is attributed to the localised charge imbalance on the pendant carboxylate groups, which is resulted from the concerted interaction between pendant carboxylate groups, Pd NPs and *M*^*n*+^. This is the first example of activating pendant carboxylate groups in MOFs to form Lewis basic sites. Therefore, HKUST-1-R serves as an attractive starting material for the simultaneous incorporation of catalytic active metal NPs and basic sites to engineer multifunctional nanocomposites. As a proof of concept, in this work we use multistep catalytic reactions to showcase the tunable multifunctionality of Pd/*M*-HKUST-1-R.

## Results

### Characterisations of Pd/HKUST-1-R and Pd/*M*-HKUST-1-R

**Fig. 2:**
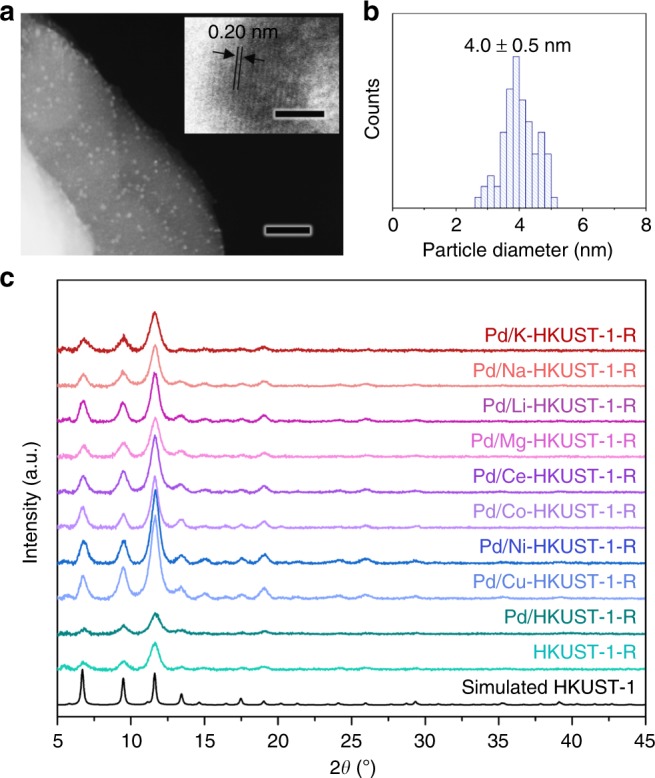
Characterisation of Pd/HKUST-1-R and its various derivatives. **a** HAADF-STEM image of Pd/HKUST-1-R with Pd loading of 1.0 wt%; TEM image of a Pd NP at high magnification in the inset. **b** Size distribution histogram of the Pd NPs supported on HKUST-1-R. **c** PXRD patterns of HKUST-1-R and its derived composite materials. Scale bars: **a** 50 nm and 2 nm (inset)

Transmission electron microscopic (TEM) image clearly shows that Pd NPs in Pd/HKUST-1-R are evenly distributed on the host support and they have a uniform size distribution of 4.0 nm (Fig. 2a, b). The (200) lattice fringes of face centred cubic Pd are observed as shown in the inset. At the same time, the hierarchical ring-like structure of the MOF support is well preserved after the deposition of Pd NPs (Supplementary Fig. 2). The powdered X-ray diffraction (PXRD) pattern of Pd/HKUST-1-R matches well with simulated HKUST-1 diffraction pattern, though no distinct peak of Pd crystal phase is observed owing to the ultra-small dimension of the Pd NPs. As mentioned above, residual CTA^+^ ions imprinted at the defect sites within Pd/HKUST-1-R are able to undergo cation exchange with various *M*^*n*+^ (*M*^*n*+^ = Ce^3+^, Co^2+^, Ni^2+^, Cu^2+^, Mg^2+^, Li^+^, Na^+^ or K^+^) to form Pd/*M*-HKUST-1-R. Similar to our previous work^30^, CTA^+^ ions are removed and the exchanged cations are uniformly distributed within the MOF structure while retaining their crystal structure (Fig. 2c and Supplementary Figs. 3 and 4). The elemental compositions of the products are summarised in Supplementary Table 1. At the same time, hierarchically porous nature of Pd/*M*-HKUST-1-R is attained after the replacement of CTA^+^ with *M*^*n*+^ (Supplementary Fig. 5 and Supplementary Table 2) because of a vast difference in size between CTA^+^ and *M*^*n*+^.

### Growth of Pd NPs in HKUST-1-R

The preparation of Pd/HKUST-1-R nanocomposite utilises a convenient in situ reduction approach, which involves a thermal treatment of HKUST-1-R and Pd^2+^ at 60 °C in ethanol. Unlike most in situ approaches to prepare metal/MOF composites^31,32^, no external reducing agent such as NaBH_4_ or H_2_ is required in this work. By inspecting the samples prepared under different conditions using TEM and X-ray photoelectron spectroscopy (XPS), it is apparent that temperature is a crucial parameter to reduce Pd^2+^ (Supplementary Fig. 6). To further understand the process of this reduction process, we analysed the supernatant of the product mixture using gas chromatography-mass spectrometry (GC-MS; Supplementary Fig. 7). Acetaldehyde was detected in the solution, which suggests that ethanol is responsible for reducing Pd^2+^ as it oxidises to its corresponding aldehyde. Reducing Pd^2+^ through heating in alcohol had been reported previously but surfactants were necessary to achieve ultra-small Pd NPs that are <5 nm^33^. As a control, we carried out the reduction of Pd^2+^ in ethanol without HKUST-1-R and with Cu-HKUST-1-R, i.e. Cu^2+^-exchanged HKUST-1-R (Supplementary Fig. 8). Clearly, CTA^+^ surfactant imprinted within HKUST-1-R is necessary to keep the particle size small and uniform. We hypothesise that, after CTA^+^ in HKUST-1-R undergoes a cation exchange with Pd^2+^, it also acts as a capping agent and restricts the growth of Pd NPs. Owing to the reduction of Pd^2+^ to Pd^0^, it is expected that expelled CTA^+^ in the solution will return back to the MOF to balance the negative charges on the pendant carboxylate groups (Supplementary Fig. 3). Consequently, Pd/HKUST-1-R is still able to further carry out cation exchange with other metal ions. We have also extended this facile approach to prepare Ag/HKUST-1-R and alloyed AgPd/HKUST-1-R by simply changing the metal precursors used (Supplementary Figs. 9–12).

### Creation of Lewis basic sites in Pd/*M*-HKUST-1-R

The ease of replacing CTA^+^ with *M*^*n*+^ (where *M*^*n*+^ = Ce^3+^, Co^2+^, Ni^2+^, Cu^2+^, Mg^2+^, Li^+^, Na^+^, or K^+^) in Pd/HKUST-1-R via cation exchange suggests that it is possible to tune the electronic state of the oxygen atoms on the pendant carboxylate groups. If the electron density of the oxygen atoms can be increased, they can potentially act as Lewis basic sites. To detect the existence of Lewis basic sites, we performed a model reaction for Lewis basic catalysts, i.e. Knoevenagel condensation. As presented in Supplementary Table 3, Pd/Cu-HKUST-1-R only yielded acetals due to the presence of Lewis acid sites that catalyse the reaction between benzaldehyde and ethanol. Interestingly, Na^+^-exchanged Pd/Na-HKUST-1-R achieved almost quantitative yield of Product **2**, which clearly indicates the presence of Lewis basic sites. On the other hand, negligible basic sites are present for MOF catalysts without Pd NPs, namely Cu-HKUST-1-R and Na-HKUST-1-R. These comparisons suggest that the source of Lewis basicity does not originate from oxide impurity formed from the addition of Na^+^. Instead, we propose that the presence of both Pd NPs and appropriate *M*^*n*+^ is essential for the creation of Lewis basic sites.

**Fig. 3:**
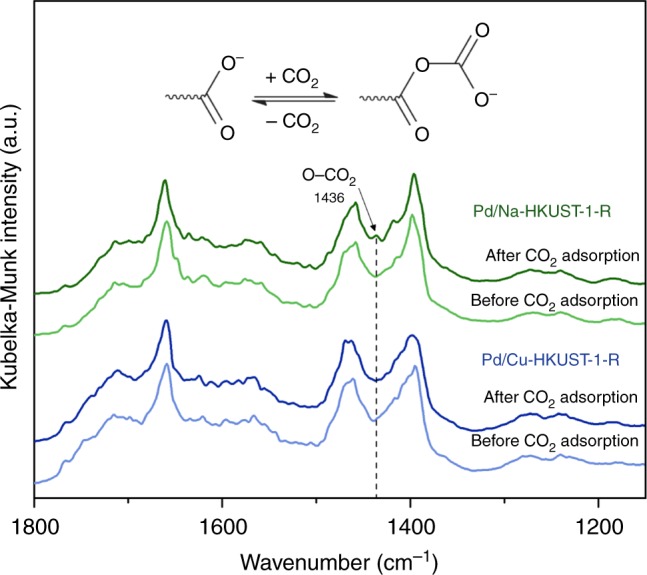
Comparison of DRIFT spectra of Pd/Na-HKUST-1-R and Pd/Cu-HKUST-1-R. DRIFT spectroscopic measurements of both samples were conducted before and after CO_2_ adsorption. The formation of carbonate ions in Pd/Na-HKUST-1-R was observed in the presence of CO_2_

Diffuse reflectance infrared Fourier transformed (DRIFT) spectroscopy of the adsorbed CO_2_ was performed to identify the Lewis basic sites present in Pd/*M*-HKUST-1-R (Fig. 3). By comparing Pd/Cu-HKUST-1-R and Pd/Na-HKUST-1-R, an additional infrared band at 1436 cm^−1^ is observed when Na^+^ is used as the charge-balancing cation. This new characteristic peak can be assigned to the asymmetrical stretching vibration of the carbonate ion due to the O−CO_2_ interaction^34^. The interaction between CO_2_ and carboxylate group herein is similar to a recent report by Chen et al., in which they showed that appropriate activation of the carboxylate groups in an ionic liquid could increase their Lewis basicity^34^. In our work, intriguingly, the presence of Pd NPs and appropriate selection of *M*^*n*+^ counterions are able to activate the pendant carboxylate groups to generate Lewis basic sites.

### Origin of Lewis basicity in Pd/*M*-HKUST-1-R

**Fig. 4:**
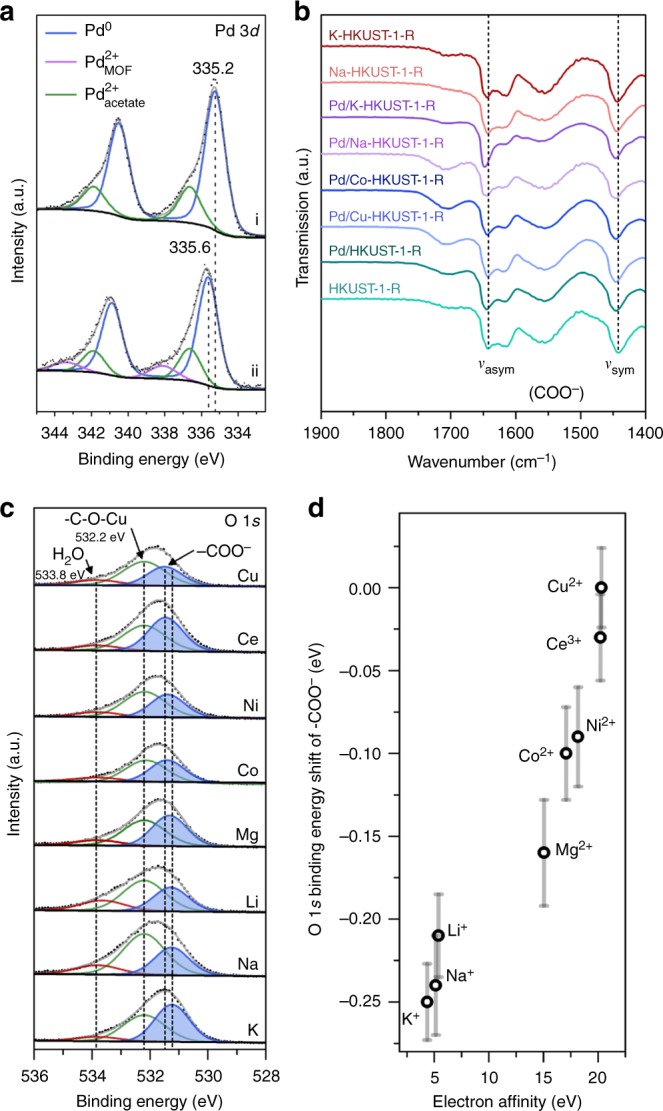
Spectroscopic characterisation of Pd/HKUST-1-R and Pd/*M*-HKUST-1-R. **a** XPS Pd 3*d* spectra of (i) bare Pd NPs (Supplementary Fig. 8) and (ii) Pd/HKUST-1-R. **b** FTIR spectra of HKUST-1-R and its various derived products. **c** XPS O 1*s* spectra of the various cation-exchanged Pd/*M*-HKUST-1-R. **d** Shift in O 1*s* binding energy with respect to the EA of the counterions. The error bars represent the standard deviations of the data points (*n* = 3)

To investigate the mechanism behind the activation of the pendant carboxylate groups, we characterised the change in chemical and electronic state of the carboxylate species by performing XPS and Fourier transform infrared (FTIR) spectroscopic measurements (Fig. 4). By comparing the Pd 3*d* spectra of bare Pd NPs and Pd/HKUST-1-R, a significant upward shift of binding energy (BE) is observed for the latter (Fig. 4a). This indicates a lower electron density on the Pd NPs supported on HKUST-1-R, which suggests the transfer of electrons from the metal to the MOF support^19,35^. This electron transfer is also observed in our FTIR investigation (Fig. 4b). For Pd/HKUST-1-R, a slight blue shift for both symmetric and asymmetric vibrational frequencies (*ν*_sym_ and *ν*_asym_) of carboxylate anion is evident when compared to HKUST-1-R. This proves a strong metal−support interaction, which involves the electron transfer from the electron-rich Pd atoms to the proximal carboxylate groups^31^. Interestingly, the blue shift in the two vibrational modes is more prominent for Pd/Na-HKUST-1-R and Pd/K-HKUST-1-R, which indicates even stronger carboxylate bonds and higher electron density localised on the carboxylate groups. However, Na-HKUST-1-R and K-HKUST-1-R (without embedded Pd NPs) show no shift in vibrational band, thus reaffirming that the source of charge transfer is the Pd NPs while the cations (*M*^*n*+^) used have significant effects of the overall charge transfer (Fig. 4b).

The effect of *M*^*n*+^ used for cation exchange is further studied with XPS technique. As shown in Fig. 4c, deconvoluted spectra of O 1*s* mainly consist of three oxygen species. The peak at 532.2 eV can be assigned to the unperturbed carboxylate groups taking part in the paddle-wheel building units (C−O−Cu)^36^. A weak shoulder peak at 533.8 eV represents trace amount of H_2_O adsorbed at the unsaturated copper sites. Lastly, the O 1*s* peak ranging from 531.2 to 531.5 eV are assigned to the pendant carboxylate groups (COO^−^) associated with the charge-balancing species. As the charge-balancing species vary from Cu^2+^ to K^+^, a negative shift of the O 1*s* BE is observed. This negative shift in BE indicates an increase in electron density on the oxygen of pendant carboxylate groups, which is in good agreement with the results presented in Fig. 4b. This phenomenon is possibly attributed to the electron affinity (EA) of *M*^*n*+^ counterions^37^. Clearly, there is a strong correlation between the EA of *M*^*n*+^ and the BE of O atoms in the pendant carboxylate groups, i.e. *M*^*n*+^ with higher EA result in higher BEs correspondingly (Fig. 4d). A possible explanation is that the negative charges of the oxygen atoms are lowered and stabilised when electron-withdrawing cations are used. The opposite happens for cations with lower EA (i.e. Mg^2+^, Li^+^, Na^+^ and K^+^), which results in oxygen atoms with higher electron density.

These observations are consistent with the hard/soft acid/base principle (Supplementary Fig. 13)^38^. Since Mg^2+^, Li^+^, Na^+^ and K^+^ are classified as hard acids while carboxylate groups are classified as hard bases, π-electron transfer from the ligand to these metal ions is minimal. Therefore, the electron density on the pendant carboxylate groups enhanced by Pd NPs can be retained. On the other hand, transition metal ions used in this study are classified as borderline acids. This means that there is a higher degree of electron transfer between the ligands and the metal ions, which lowers the electron density on the pendant carboxylate groups. Indeed, these electron transfer behaviours are observed from additional XPS analysis of *M*-HKUST-1-R and Pd-*M*-HKUST-1-R (Supplementary Figs. 14 and 15), which are consistent with the hard/soft acid/base theory and the observed changes in electron densities of O atoms on the pendant carboxylate groups (Fig. 4c, d).

The results presented in Fig. 4 reveal two important findings. First, Pd NPs interact strongly with HKUST-1-R support through electron donation towards the pendant carboxylate groups. Second, the use of *M*^*n*+^ with low EA allow the preservation of high electron density on the pendant carboxylate groups. Hence, the cooperative interactions of Pd‒pendant carboxylate groups‒*M*^*n*+^ resulted in the unbalanced charge localisation on the pendant carboxylate groups. Ultimately, such charge localisation enables the activation of pendant carboxylate groups to act as Lewis basic sites, thus allowing Pd/Na-HKUST-1-R to catalyse Knoevenagel condensation successfully (Supplementary Table 3). Knoevenagel condensation was also performed using Pd_*y*%_/Na-HKUST-1-R of different Pd loadings, where *y*% = wt% of Pd (Supplementary Fig. 16). Theoretically, since these discrete Lewis basic sites are located in close proximity to the Pd NPs, the density of the Lewis basic sites should be proportional to the Pd content in Pd/Na-HKUST-1-R. Indeed, Pd_0.5%_/Na-HKUST-1-R and Pd_1.0%_/Na-HKUST-1-R attain similar catalytic reaction profile, since the amount of catalysts used was fixed based on the mole of Pd, i.e. the density of Lewis basic sites in Pd_0.5%_/Na-HKUST-1-R is half of Pd_1.0%_/Na-HKUST-1-R (Supplementary Fig. 17). However, Pd_2.6%_/Na-HKUST-1-R exhibit a poorer catalytic performance, which suggests that there is a saturation limit to the density of Lewis basic sites.

### Pd NP-enhanced Knoevenagel condensation

**Fig. 5:**
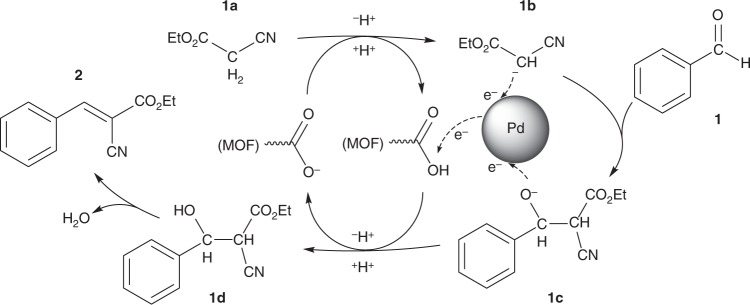
Proposed mechanism of Pd^0^-enhanced base-catalysed Knoevenagel condensation. The Pd NP acts as an electron reservoir to stabilise anionic intermediates (**1b** and **1c**) and facilitates the regeneration of MOF Lewis basic sites via electron transfer

Another possible role of Pd NPs in the condensation reaction is their ability to behave as an electron reservoir^39^. As presented in Fig. 5, we propose a modified reaction mechanism for Pd/Na-HKUST-1-R-catalysed Knoevenagel condensation. From the reaction scheme, the pendant carboxylate Lewis basic site is involved in the deprotonation of methylene group in ethyl cyanoacetate **1a** to form a carbanion **1b**. The Lewis basic site is then regenerated by protonating the anionic intermediate **1c** to form a hydroxyl-containing intermediate **1d**. The presence of Pd NPs could stabilise the anion **1b** by partially accepting the lone pair of electrons. As a result, protonation of **1b** back to **1a** can be minimised. Similarly, it is also possible that benzaldehyde molecule **1** is favourably adsorbed onto the surface of Pd NP due to its electron-rich carbonyl groups. Hence, **1** and **1b** can be brought together in close proximity for nucleophilic addition step to occur. Second, the Pd NP could also facilitate electron transfer from the anion intermediate **1c** to the protonated Lewis basic site, thus assisting the regeneration of the basic site. Therefore, in addition to playing a part in the creation of Lewis basic sites, Pd NPs can facilitate the Knoevenagel condensation by acting as a co-catalyst.

### Multifunctionality of Pd/*M*-HKUST-1-R

The presence of catalytic active Pd NPs, inherent Lewis acid sites and newly engineered Lewis basic sites encourages us to explore the practicality of the multifunctional Pd/*M*-HKUST-1-R. As a simple demonstration, we performed a multistep catalytic reaction, specifically oxidation–Knoevenagel condensation reactions (Table 1). First, Pd NPs catalyse the oxidation of benzyl alcohol to benzaldehyde, **1**. Second, the Lewis basic sites catalyse the condensation of ethyl cyanoacetate and **1** to form **2**. In addition, by performing the above reactions in ethanol, acetalisation between **1** and ethanol can also be catalysed by the acid sites in the MOF support to form acetal, **3**. Both **2** and **3** have their industrial importance; **2** is an important intermediate for fragrant and pharmaceutical products, while **3** is a useful form to protect the carbonyl group of aldehydes. Hence, it is desirable if product selectivity can be controlled through rational design of catalysts. This can be achieved by tuning the Lewis basicity in Pd/*M*-HKUST-1-R. In the absence of basic sites, only **3** will be formed from **1**. In the presence of adequate Lewis basic sites, **2** will be the major product due to the reversible nature of acetal formation.

**Table 1:** Pd/*M*-HKUST-1-R catalysed stepwise oxidation^a^–Knoevenagel condensation^b^ reactions


Bi-catalysts	Yield (%)		
	1	2	3
Pd/HKUST-1-R^c^	11.6	35.0	0.8
Pd/Cu-HKUST-1-R	9.0	0.0	91.0
Pd/Ce-HKUST-1-R	6.8	1.2	92.0
Pd/Ni-HKUST-1-R	19.8	26.1	54.1
Pd/Co-HKUST-1-R	20.5	47.3	32.2
Pd/Mg-HKUST-1-R	7.0	90.6	2.4
Pd/Li-HKUST-1-R	4.2	95.0	0.8
Pd/Na-HKUST-1-R	1.4	98.1	0.5
Pd/K-HKUST-1-R	8.1	91.9	0.0

^a^Reaction conditions: benzyl alcohol (1 mmol), ethanol (10 mL), Pd/M-HKUST-1-R (0.5 mol% Pd), 75 °C, in flowing O_2_ gas (1 atm), 20 h

^b^Reaction conditions: ethyl cyanoacetate (1.5 mmol), room temperature (22 °C) and pressure, 24 h

^c^Only 47.4% conversion were achieved for benzyl alcohol oxidation using this control sample. The formation of **3** occurs throughout both oxidation and condensation reactions. Functional catalytic components of Pd/*M*-HKUST-1-R in the above three reactions are indicated in red

For all of the prepared Pd/*M*-HKUST-1-R nanocatalysts, 100% conversion of benzyl alcohol can be achieved without the formation of benzoic acid (Supplementary Table 4). After further reaction with ethyl cyanoacetate for 24 h at room temperature, various product distributions can be obtained depending on *M*^*n*+^ grafted within Pd/*M*-HKUST-1-R. As expected, the use of cations with low EA, such as Mg^2+^, Li^+^, Na^+^ and K^+^, provides high yields of **2** and negligible yield of **3**. This reveals that these MOF supports are dominated by strong Lewis basic sites. It is worth noting that even though K^+^ has the lowest EA, i.e. strongest basicity, Pd/K-HKUST-1-R achieves a lower yield of **2** than Pd/Na-HKUST-1-R. This could be attributed to the larger ionic radius of K^+^ compared to Na^+^, thus causing partial steric hindrance to the active Lewis basic sites. As such, this could have caused Pd/K-HKUST-1-R to exhibit lower surface area as compared to Pd/Na-HKUST-1-R (Supplementary Table 2). On the other hand, Pd/Cu-HKUST-1-R yields no Product **2**, which suggests the absence of basic site. As shown in our previous work^30^, the addition of Cu^2+^ to Pd/HKUST-1-R allows the healing of the defective sites, which can be interpreted by the sharpening of their PXRD peaks (Fig. 2c). As a result, Pd/Cu-HKUST-1-R would contain the highest density of unsaturated copper sites with Lewis acid properties and the lowest pendant carboxylate Lewis basic sites. Lastly, Pd/*M*-HKUST-1-R with Co^2+^, Ni^2+^ and Ce^3+^ charge-balancing cations yield a mixture of **2** and **3**, which showcase their bifunctional Lewis acid and Lewis basic properties. Since the product yields of **2** and **3** vary according to the EA of *M*^*n*+^, the above results reaffirm the hypothesised relationship between the EA of *M*^*n*+^ and the resultant Lewis basicity of Pd/*M*-HKUST-1-R. Therefore, selective production of **2** or **3** can be achieved facilely by controlling *M*^*n*+^ used in Pd/*M*-HKUST-1-R, which directly affects the Lewis acidity/basicity of the catalysts. The differences in the strength of basic sites present in Pd/*M*-HKUST-1-R as interpreted from the catalytic results are also consistent with the measurements determined by DRIFT spectroscopy of pyrrole adsorption (Supplementary Fig. 18). Moreover, Pd/Na-HKUST-1-R is able to undergo five cycles of oxidation–Knoevenagel condensation reactions without any significant drop in product yield (Supplementary Fig. 19). The high catalytic stability of the catalyst can be achieved as Pd NPs within the spent catalyst do not suffer from leaching or aggregation during the catalysis, while our PXRD measurement shows that the crystallinity of the MOF support is well preserved after the repeated catalytic reactions (Supplementary Figs. 20 and 21). It is worth noting that, when the parent MOF composite Pd/HKUST-1-R is used as the catalyst, lower yields of **1**–**3** are attained, i.e. lower conversion of benzyl alcohol. This is attributed to the low surface area and the least Lewis basicity of Pd/HKUST-1-R since cation exchange was not carried out to remove bulky CTA^+^ present in the pores (Supplementary Fig. 5 and Supplementary Table 2), which results in a lower amount of exposed active sites for catalysis.

## Discussion

In this work, a unique synthetic method to create Lewis basic sites in MOF is presented. The cooperative interactions between Pd NPs, *M*^*n*+^ (*M*^*n*+^ = Ce^3+^, Co^2+^, Ni^2+^, Cu^2+^, Mg^2+^, Li^+^, Na^+^, or K^+^) and pendant carboxylate groups in the defective anionic HKUST-1-R result in localised charge imbalance on the pendant carboxylate groups. The high electron density on these pendant carboxylate groups caused them to exhibit Lewis basicity. The level of electron density, and thus the strength of these Lewis basic sites, are correlated to the EA of *M*^*n*+^, which can be regulated with ease via cation exchange. Consequently, the synthetic strategy established here enables rational design of multifunctional MOF-based nanocomposites with tunable Lewis basicity and high stability for multistep catalysis. With the abundance of metal–carboxylate MOFs and methods to introduce defect sites reported to date, we expect our synthetic approach to impart extrinsic functionalities in this class of materials to be widely applicable.

## Methods

### Synthesis of HKUST-1-R

The synthetic procedure was reported in our previous work^30^. In general, 1.13 mL of aqueous 0.5 M Cu(NO_3_)_2_ solution and 48.0 mL of aqueous 0.1 M CTAB solution were added to 30.0 mL of deionised water. This mixture was subsequently stirred for 5 min before instantaneous addition of 80.0 mL of aqueous 0.011 M BTC^3−^ solution. The reaction solution was left to stir continuously for 30 min. The product was then collected, centrifuged and washed for 4 cycles using ethanol and later suspended in 10.0 mL of ethanol. Note that 0.011 M BTC^3−^ solution was prepared by dissolving 0.925 g H_3_BTC in 40.0 mL deionised water and 1.67 mL TEA via ultrasonication (resulting pH: 4.70), and the mixture was then diluted with 360.0 mL of deionised water.

### Synthesis of Pd/HKUST-1-R

First, 10.0 mL of HKUST-1-R ethanolic suspension was added to 80.0 mL of ethanol and this mixture was allowed to stir for 5 min. Subsequently, 3.20 mL of 5 mM Pd(OAc)_2_ acetone solution was added dropwise over 1 min. The mixture was left to stir for another 5 min before transferring into an oven controlled at 60 °C. After 12 h of reaction, the product was then collected, centrifuged and washed for 4 cycles using ethanol and later suspended in 10.0 mL of ethanol.

### Synthesis of Pd/*M*-HKUST-1-R

For Pd/Cu-HKUST-1-R, 10.0 mL of Pd/HKUST-1-R ethanolic suspension was added to 80.0 mL of ethanol and this mixture was allowed to stir for 5 min. Subsequently, 10.0 mL of methanolic 50 mM Cu(NO_3_)_2_ solution was added dropwise over 1 min. The mixture was left to stir at room conditions (22 °C) for another 3 h. The product was then collected, centrifuged and washed for 4 cycles using ethanol and later dried in an oven at 60 °C. For *M*^*n*+^ = Ce^3+^, Co^2+^, Ni^2+^, Mg^2+^, Li^+^, Na^+^ or K^+^, the Cu(NO_3_)_2_ solution was replaced with Ce(NO_3_)_3_, Co(NO_3_)_2_, Ni(NO_3_)_2_, Mg(OAc)_2_, Li(OAc), Na(OAc) or KCl solutions, respectively.

### Evaluation of oxidation–Knoevenagel condensation reactions

Pd/*M*-HKUST-1-R (0.5 mol% Pd w.r.t. benzyl alcohol) was added to a mixture of ethanol (10.0 mL) and *n*-dodecane (1.0 mmol) in a round bottom flask. This suspension was sonicated for 1 min to disperse the catalyst uniformly. The mixture was then heated in an oil bath at 75 °C while stirring with a reflux condenser and under O_2_ flow (1 atm, 10 mL min^−1^). Once the catalyst mixture reached 75 °C, benzyl alcohol (1 mmol) was injected immediately. After reaction for 20 h, the reaction mixture was transferred out of the oil bath and into a glass vial. Once the reaction mixture had cooled down to room temperature (22 °C), ethyl cyanoacetate (1.5 mmol) was added and the mixture was further stirred at room temperature for 24 h. Lastly, the solid catalyst was separated via centrifugation and the resultant supernatant was analysed by GC (Agilent-7890A) equipped with a capillary column (HP-5, 30.0 m × 320 μm × 0.25 μm) and a flame ion detector. The identities of the products were further verified by GC-MS (Agilent-7890A-5975C) equipped with a capillary column (DB-5 ms, 30.0 m × 320 μm × 0.25 μm).

### Materials' characterisation

The morphological features of the nanoparticles were investigated using TEM (JEM-2010, FETEM-2100F, accelerating voltage: 200 kV). Elemental distribution of the nanomaterials was analysed by energy dispersive X-ray spectroscopy coupled to the field emission TEM operating in high-angle annular dark-field imaging. The bulk morphology of samples were analysed with scanning electron microscopy (field emission SEM, JEM-6700F, accelerating voltage: 15 kV, working distance: 15 mm). The crystallographic structure was determined by X-ray diffractometer (Bruker D8 Advance) equipped with Cu Kα radiation source. Specific surface area of the samples were obtained from N_2_ physisorption isotherms at 77 K after overnight activation at 150 °C in N_2_ atmosphere (Quantachrome NOVA-3000 system). The organic groups present in HKUST-1-R, *M*-HKUST-1-R and Pd/*M*-HKUST-1-R were characterised by FTIR spectroscopy using ATR mode (Bruker). XPS (AXIS-HSi, Kratos Analytical) analysis was performed using a monochromatised Al *K*_α_ exciting radiation (*hν* = 1286.71 eV). The measured BEs were referenced according to C 1*s* peak (BE set at 284.5 eV) that corresponds to C−C bonds. Metal content of Pd/*M*-HKUST-1-R was analysed by inductively coupled plasma optical emission spectrometry (Optima 7300DV, Perkin Elmer). DRIFT spectroscopic measurements were carried out using Bruker TENSOR II FTIR spectrometer equipped with an MCT detector. For CO_2_ adsorption study, the powdered sample was loaded into the DRIFT cell and pretreated in N_2_ flow (50 mL min^−1^) at 150 °C for 1 h. The cell is then cooled to 25 °C and exposed to CO_2_ flow (50 mL min^−1^) for 1 h before purging with N_2_ for 1 h to remove the physically adsorbed CO_2_. Finally, the FTIR spectra were recorded.

## Electronic supplementary material

Supplementary Information

## Data Availability

The data that support the findings of this study are available from the corresponding author upon reasonable request.

## References

[1] Furukawa H, Cordova KE, O’Keeffe M, Yaghi OM (2013). The chemistry and applications of metal–organic frameworks. Science.

[2] Hendon CH, Rieth AJ, Korzynski MD, Dinca M (2017). Grand challenges and future opportunities for metal–organic frameworks. ACS Cent. Sci..

[3] Horike S, Shimomura S, Kitagawa S (2009). Soft porous crystals. Nat. Chem..

[4] Sheberla D (2017). Conductive MOF electrodes for stable supercapacitors with high areal capacitance. Nat. Mater..

[5] Farha OK (2010). De novo synthesis of a metal–organic framework material featuring ultrahigh surface area and gas storage capacities. Nat. Chem..

[6] Lee J (2009). Metal–organic framework materials as catalysts. Chem. Soc. Rev..

[7] Xiao DJ (2014). Oxidation of ethane to ethanol by N_2_O in a metal–organic framework with coordinatively unsaturated iron(II) sites. Nat. Chem..

[8] Zhu L, Liu XQ, Jiang HL, Sun LB (2017). Metal–organic frameworks for heterogeneous basic catalysis. Chem. Rev..

[9] Hasegawa S (2007). Three-dimensional porous coordination polymer functionalized with amide groups based on tridentate ligand: selective sorption and catalysis. J. Am. Chem. Soc..

[10] Li P, Zeng HC (2016). Immobilization of metal–organic framework nanocrystals for advanced design of supported nanocatalysts. ACS Appl. Mater. Inter..

[11] McDonald TM (2012). Capture of carbon dioxide from air and flue gas in the alkylamine-appended metal−organic framework mmen-Mg_2_(dobpdc). J. Am. Chem. Soc..

[12] Valvekens P, Vandichel M, Waroquier M, Van Speybroeck V, De Vos D (2014). Metal-dioxidoterephthalate MOFs of the MOF-74 type: microporous basic catalysts with well-defined active sites. J. Catal..

[13] Valvekens P (2014). Base catalytic activity of alkaline earth MOFs: a (micro)spectroscopic study of active site formation by the controlled transformation of structural anions. Chem. Sci..

[14] An J, Rosi NL (2010). Tuning MOF CO_2_ adsorption properties via cation exchange. J. Am. Chem. Soc..

[15] Quartapelle, Procopio E (2010). Cation-exchange porosity tuning in anionic metal–organic frameworks for the selective separation of gases and vapors and for catalysis. Angew. Chem. Int. Ed..

[16] Park SS, Tulchinsky Y, Dinca M (2017). Single-ion Li^+^, Na^+^, and Mg^2+^ solid electrolytes supported by a mesoporous anionic Cu-azolate metal–organic framework. J. Am. Chem. Soc..

[17] Lu G (2012). Imparting functionality to a metal–organic framework material by controlled nanoparticle encapsulation. Nat. Chem..

[18] Yang Q, Xu Q, Jiang HL (2017). Metal‒organic frameworks meet metal nanoparticles: synergistic effect for enhanced catalysis. Chem. Soc. Rev..

[19] Zhao M (2016). Metal‒organic frameworks as selectivity regulators for hydrogenation reactions. Nature.

[20] Zhan G, Zeng HC (2016). Integrated nanocatalysts with mesoporous silica/silicate and microporous MOF materials. Coord. Chem. Rev..

[21] Zeng HC (2013). Integrated nanocatalysts. Acc. Chem. Res..

[22] Zhan G, Zeng HC (2017). Smart nanocatalysts with streamline shapes. ACS Cent. Sci..

[23] Li Z, Zeng HC (2013). Surface and bulk integrations of single-layered Au or Ag nanoparticles onto designated crystal planes (110) or (100) of ZIF-8. Chem. Mater..

[24] Xi B, Tan YC, Zeng HC (2015). A general synthetic approach for integrated nanocatalysts of metal-silica@ZIFs. Chem. Mater..

[25] Yang Q, Xu Q, Yu SH, Jiang HL (2016). Pd nanocubes@ZIF-8: integration of plasmon-driven photothermal conversion with a metal‒organic framework for efficient and selective catalysis. Angew. Chem. Int. Ed..

[26] Chen YZ (2017). Singlet oxygen-engaged selective photo-oxidation over Pt nanocrystals/porphyrinic MOF: the roles of photothermal effect and Pt electronic state. J. Am. Chem. Soc..

[27] Fang Z, Bueken B, De Vos DE, Fischer RA (2015). Defect-engineered metal‒organic frameworks. Angew. Chem. Int. Ed..

[28] Bennett TD, Cheetham AK, Fuchs AH, Coudert FX (2016). Interplay between defects, disorder and flexibility in metal‒organic frameworks. Nat. Chem..

[29] Tan YC, Zeng HC (2016). Self-templating synthesis of hollow spheres of MOFs and their derived nanostructures. Chem. Commun..

[30] Tan YC, Zeng HC (2017). Defect creation in HKUST-1 via molecular imprinting: attaining anionic framework property and mesoporosity for cation exchange applications. Adv. Funct. Mater..

[31] Chen L, Chen H, Luque R, Li Y (2014). Metal‒organic framework encapsulated Pd nanoparticles: towards advanced heterogeneous catalysts. Chem. Sci..

[32] Jiang HL, Akita T, Ishida T, Haruta M, Xu Q (2011). Synergistic catalysis of Au@Ag core-shell nanoparticles stabilized on metal-organic framework. J. Am. Chem. Soc..

[33] Wang X, Zhuang J, Peng Q, Li Y (2005). A general strategy for nanocrystal synthesis. Nature.

[34] Chen FF (2016). Multi-molar absorption of CO_2_ by the activation of carboxylate groups in amino acid ionic liquids. Angew. Chem. Int. Ed..

[35] Li G (2014). Hydrogen storage in Pd nanocrystals covered with a metal‒organic framework. Nat. Mater..

[36] Kozachuk O (2011). Solvothermal growth of a ruthenium metal‒organic framework featuring HKUST-1 structure type as thin films on oxide surfaces. Chem. Commun..

[37] Pearson RG (1988). Absolute electronegativity and hardness: application to inorganic chemistry. Inorg. Chem..

[38] Pearson RG (1963). Hard and soft acids and bases. J. Am. Chem. Soc..

[39] Ye M, Gong J, Lai Y, Lin C, Lin Z (2012). High-efficiency photoelectrocatalytic hydrogen generation enabled by palladium quantum dots-sensitized TiO_2_ nanotube arrays. J. Am. Chem. Soc..

