# Supplementary material for: Lewis basicity generated by localised charge imbalance in noble metal nanoparticle-embedded defective metal–organic frameworks
Source: Nat Commun. 2018 Oct 18;9:4326. doi: 10.1038/s41467-018-06828-4 (PMC6194069; doi:10.1038/s41467-018-06828-4)
Supplement: Supplementary file 1 — Supplementary Information [file 41467_2018_6828_MOESM1_ESM.pdf]

Supplementary Information

Lewis basicity generated by localised charge imbalance in noble metal nanoparticle-embedded defective metal–organic frameworks

Ying Chuan Tan[1,2] and Hua Chun Zeng[1,2,*]

[1]*Department of Chemical and Biomolecular Engineering, Faculty of Engineering, National University of Singapore, 10 Kent Ridge Crescent, Singapore 119260.* [2]*Cambridge Centre for Advanced Research in Energy Efficiency in Singapore, 1 Create Way, Singapore 138602, Singapore*

*E-mail: chezhc@nus.edu.sg

Contents

Supplementary Methods: Page 2 to Page 4

Supplementary Figures: Page 4 to Page 22

Supplementary Tables: Page 23 to Page 24

Supplementary References: Page 25

Supplementary Methods

Materials. The following chemicals were used as received without further purification. Methanol (VWR Chemicals, analytical reagent grade), ethanol (VWR Chemicals, analytical reagent grade), acetone (VWR Chemicals, analytical reagent grade), hexadecyltrimethylammonium bromide (CTAB, Fluka, >96.0%), palladium (II) acetate ($Pd(OAc)_2$, Sigma-Aldrich, 98%), silver (I) nitrate ($AgNO_3$, Sigma-Aldrich, ≥99.0%) copper (II) nitrate trihydrate ($Cu(NO_3)_2$, Sigma-Aldrich, ≥ 98.0%), trimesic acid (H_3BTC, Sigma-Aldrich, 95.0%), triethylamine (TEA, Merck, ≥99.0%), cerium (III) nitrate hexahydrate ($Ce(NO_3)_3$, Sigma-Aldrich, ≥99.0%), cobalt (II) nitrate hexahydrate ($Co(NO_3)_2$, Sigma-Aldrich, ≥ 98.0%), potassium chloride (KCl, Merck, ≥99.5%), lithium acetate (Li(OAc), Sigma-Aldrich, ≥99.95%), magnesium (II) acetate tetrahydrate ($Mg(OAc)_2$, Fluka, ≥99.0%), sodium acetate anhydrous (Na(OAc), Fluka, ≥99.0%), nickel (II) nitrate hexahydrate ($Ni(NO_3)_2$, Merck, ≥99.0%), benzaldehyde (Sigma-Aldrich, 99.0%), benzyl alcohol (Alfa Aesar, 99.0%), ethyl cyanoacetate (Sigma-Aldrich, 98.0%).

Synthesis of HKUST-1-R. The synthetic procedure was reported in our previous work[1]. In general, 1.13 mL of aqueous 0.5 M $Cu(NO_3)_2$ solution and 48.0 mL of aqueous 0.1 M CTAB solution were added to 30.0 mL of deionised water. This mixture was subsequently stirred for 5 min before instantaneous addition of 80.0 mL of aqueous 0.011 M BTC^{3-} solution. The reaction solution was left to stir continuously for 30 min. The product was then collected, centrifuged and washed for 4 cycles using ethanol and later suspended in 10.0 mL ethanol. Note that 0.011 M BTC^{3-} solution was prepared by dissolving 0.925 g H_3BTC in 40.0 mL deionised water and 1.67 mL TEA via ultrasonication (resulting pH: 4.70), and the mixture was then diluted with 360.0 mL of deionised water.

Synthesis of Pd/HKUST-1-R. First, 10.0 mL of HKUST-1-R ethanolic suspension was added to 80.0 mL of ethanol and this mixture was allowed to stir for 5 min. Subsequently, 3.20 mL of 5 mM $Pd(OAc)_2$ acetone solution was added dropwise over 1 min. The mixture was left to stir for another 5 min before transferring into an oven controlled at 60 °C. After 12 h of reaction, the product was then collected, centrifuged and washed for 4 cycles using ethanol and later suspended in 10.0 mL ethanol.

Synthesis of Ag/HKUST-1-R. The synthetic procedure is similar to that of Pd/HKUST-1-R, except the addition of 3.20 mL of 5 mM $Pd(OAc)_2$ acetone solution is replaced by 4.00 mL of 10 mM $AgNO_3$ methanol solution.

Synthesis of AgPd/HKUST-1-R. The synthetic procedure is similar to that of Pd/HKUST-1-R, except 0.80 mL of 10 mM $AgNO_3$ methanol solution is added in addition to 3.20 mL of 5 mM $Pd(OAc)_2$ acetone solution.

Synthesis of Pd/*M*-HKUST-1-R. For Pd/Cu-HKUST-1-R, 10.0 mL of Pd/HKUST-1-R ethanolic suspension was added to 80.0 mL of ethanol and this mixture was allowed to stir for 5 min. Subsequently, 10.0 mL of methanolic 50 mM $Cu(NO_3)_2$ solution was added dropwise over 1 min. The mixture was left to stir at room conditions for another 3 h. The product was then collected, centrifuged and washed for 4 cycles using ethanol and later dried in an oven at 60 °C. For M^{n+} = Ce^{3+}, Co^{2+}, Ni^{2+}, Mg^{2+}, Li^{+}, Na^{+}, or K^{+}, the $Cu(NO_3)_2$ solution was replaced with $Ce(NO_3)_3$, $Co(NO_3)_2$, $Ni(NO_3)_2$, $Mg(OAc)_2$, Li(OAc), Na(OAc), or KCl solutions respectively.

Materials characterisation. The morphological features of the nanoparticles were investigated using transmission electron microscopy (TEM, JEM-2010, FETEM-2100F, accelerating voltage: 200 kV). Elemental distribution of the nanomaterials was analysed by energy dispersive X-ray (EDX) spectroscopy coupled to the FETEM operating in high-angle annular dark-field imaging (HAADF). The bulk morphology of samples were analysed with scanning electron microscopy (FESEM, JEM-6700F, accelerating voltage: 15 kV, working distance: 15 mm). The crystallographic structure was determined by X-ray diffractometer (XRD, Bruker D8 Advance) equipped with Cu K_α radiation source. Specific surface area and pore size distribution of samples were obtained from N_2 physisorption isotherms at 77 K after overnight activation of the samples at 150 °C in N_2 atmosphere (Quantachrome NOVA-3000 system). The organic groups present in HKUST-1-R, *M*-HKUST-1-R and Pd/*M*-HKUST-1-R were characterised by Fourier transform infrared spectroscopy using ATR mode (FTIR, Bruker). X-ray photoelectron spectroscopy (XPS, AXIS-HSi, Kratos Analytical) analysis was performed using a monochromatised Al K_α exciting radiation (hv = 1286.71 eV). The measured binding energies (BEs) were referenced according to C 1*s* peak (BE set at 284.5 eV) that corresponds to C−C bonds. Metal content of Pd/*M*-HKUST-1-R was analysed by inductively coupled plasma optical emission spectrometry (ICP-OES, Optima 7300DV, Perkin Elmer).

Diffuse reflectance infrared Fourier transformed spectroscopy (DRIFT) measurements were carried out using Bruker TENSOR II FTIR spectrometer equipped with a MCT detector. For CO_2 adsorption study, the powdered sample was loaded into the DRIFT cell and pretreated in N_2 flow (50 mL·min^{-1}) at 150 °C for 1 h. The cell is then cooled to 25 °C and exposed to CO_2 flow (50 mL·min^{-1}) for 1 h before purging with N_2 for 1 h to remove physically adsorbed CO_2. Finally, the FTIR spectra were recorded. For pyrrole adsorption study, the activated samples of Pd/*M*-HKUST-1-R were exposed to pyrrole vapour at room temperature for 3 h. Subsequently, the samples were purged in the DRIFT cell at 60 °C under N_2 flow for 5 h to remove physisorbed pyrrole molecules before recording the FTIR spectra.

Catalytic evaluation for Knoevenagel condensation reaction. Pd/*M*-HKUST-1-R (0.5 mol% Pd w.r.t. aldehyde) was added to a mixture of ethanol (10.0 mL) and *n*-dodecane (1.0 mmol) in a glass vial. This suspension was sonicated for 1 min to disperse the catalyst uniformly. Benzaldehyde (1.0 mmol) and ethyl cyanoacetate (1.5 mmol) were added simultaneously to the catalyst suspension under stirring condition to start the reaction. After 24 h, the solid catalyst was separated via centrifugation and the resultant supernatant was analysed by gas chromatography (GC, Agilent-7890A) equipped with a capillary column (HP-5, 30.0 m × 320 μm × 0.25 μm) and a flame ion detector (FID). The identities of the products were further verified by gas chromatography-mass spectrometry (GC-MS, Agilent-7890A-5975C) equipped with a capillary column (DB-5 ms, 30.0 m × 320 μm × 0.25 μm).

Catalytic evaluation for oxidation-Knoevenagel condensation reactions. Pd/*M*-HKUST-1-R (0.5 mol% Pd w.r.t. benzyl alcohol) was added to a mixture of ethanol (10.0 mL) and *n*-dodecane (1.0 mmol) in a round bottom flask. This suspension was sonicated for 1 min to disperse the catalyst uniformly. The mixture was then heated in an oil bath at 75 °C while stirring with a reflux condenser and under O_2 flow (1 atm, 10 mL·min^{-1}). Once the catalyst mixture reached 75 °C, benzyl alcohol (1.0 mmol) was injected immediately. After reacting for 20 h, the reaction mixture was transferred out of the oil bath and into a glass vial. Once the reaction mixture had cooled down to room temperature, ethyl cyanoacetate (1.5 mmol) was added and the mixture was further stirred at room temperature for 24 h. Lastly, the solid catalyst was separated via centrifugation and the resultant supernatant was analysed by GC equipped with

a capillary column and a FID. The identities of the products were further verified by GC-MS equipped with a capillary column.

Supplementary Figures

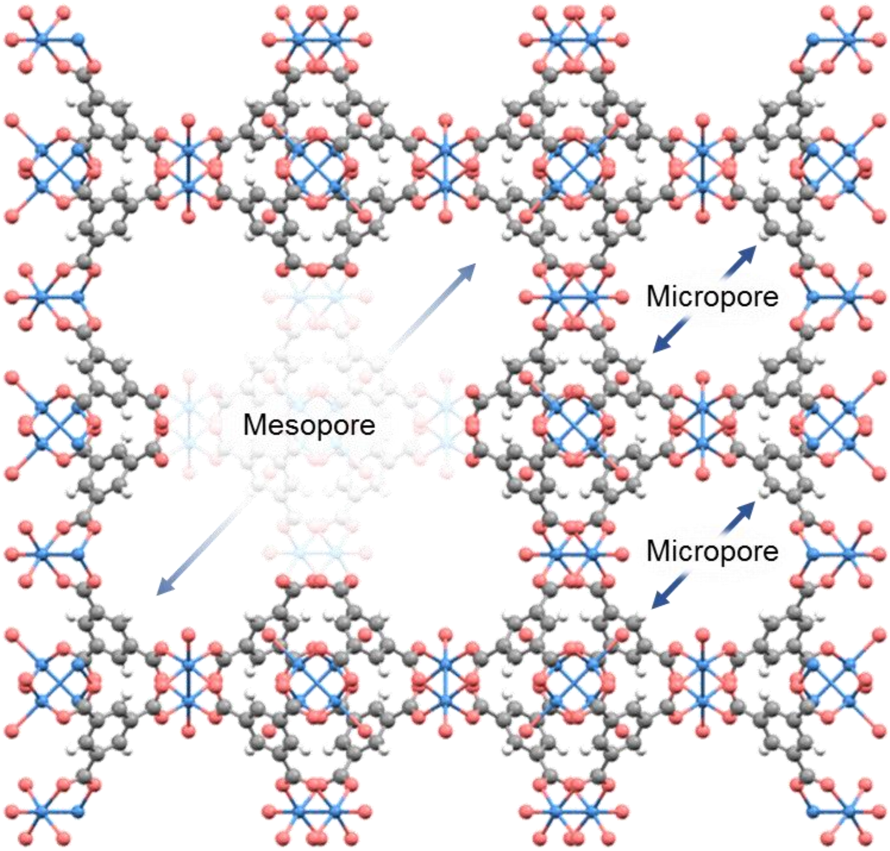

Supplementary Figure 1 Structural representation of *M*-HKUST-1-R. Extrinsic mesopores are present in *M*-HKUST-1-R due to defects resulted from metal clusters vacancies. The counterions are omitted for clarity. The C, O and H atoms of the ligands are shown as grey, red and white spheres respectively. Cu^{2+} ions are shown as blue spheres.

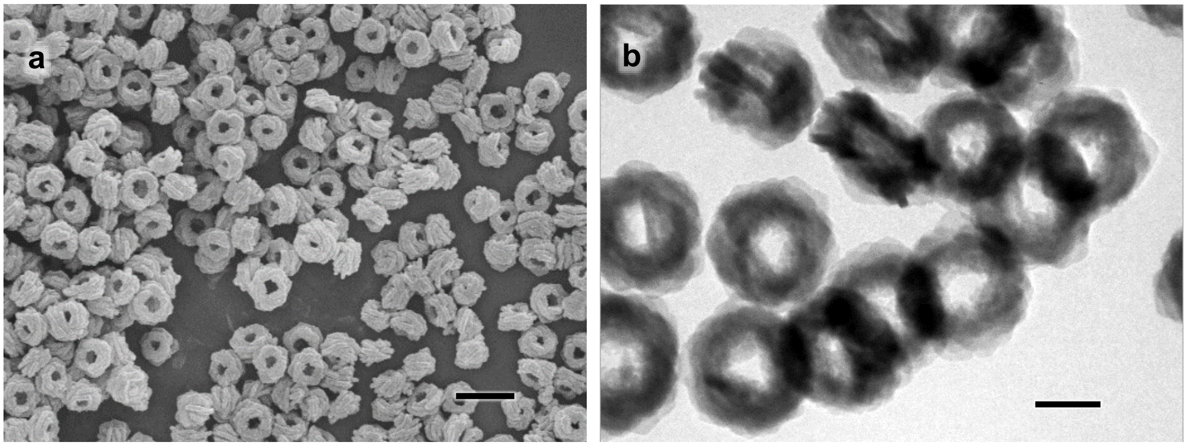

Supplementary Figure 2 Morphological property of Pd/HKUST-1-R. **a**, panoramic view of Pd/HKUST-1-R characterised using SEM. **b**, close-up view of Pd/HKUST-1-R characterised using TEM. Scale bars: **a** 2 µm, **b** 600 nm

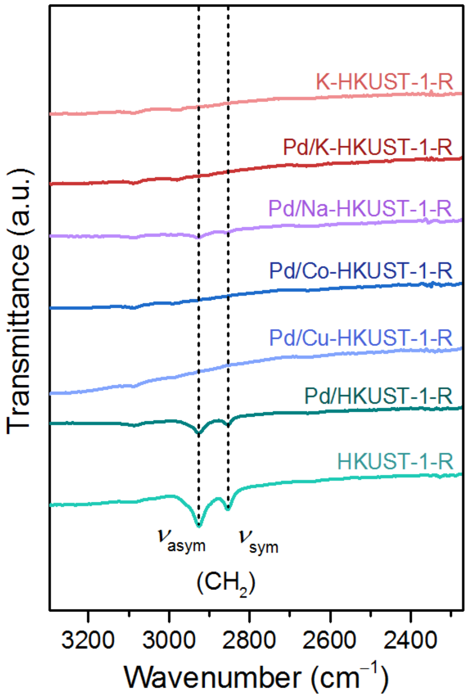

Supplementary Figure 3 FTIR spectra of HKUST-1-R and its various derived products after undergoing cation exchange. Note that the disappearance of the peaks for symmetric and asymmetric stretching modes (ν_{sym} and ν_{asym}) of CH_2 suggests that CTA^+ imprinted in HKUST-1-R are removed effectively by cation exchange.

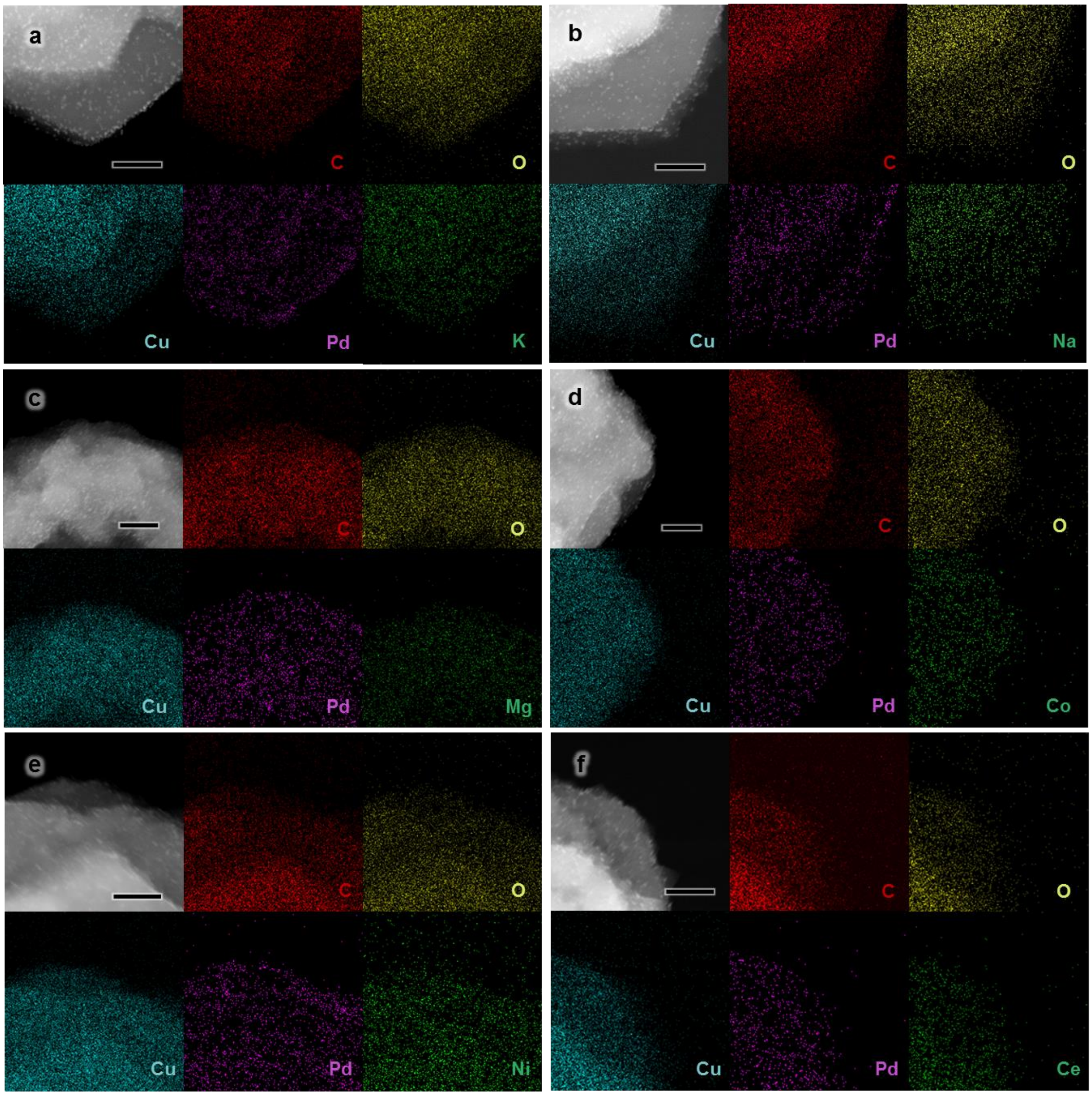

Supplementary Figure 4 Characterisation of Pd/*M*-HKUST-1-R. HAADF-STEM image of Pd/*M*-HKUST-1-R and their corresponding elemental mapping images. **a**, Pd/K-HKUST-1-R. **b**, Pd/Na-HKUST-1-R. **c**, Pd/Mg-HKUST-1-R. **d**, Pd/Co-HKUST-1-R. **e**, Pd/Ni-HKUST-1-R. **f**, Pd/Ce-HKUST-1-R. Scale bars: **a**–**f** 100 nm

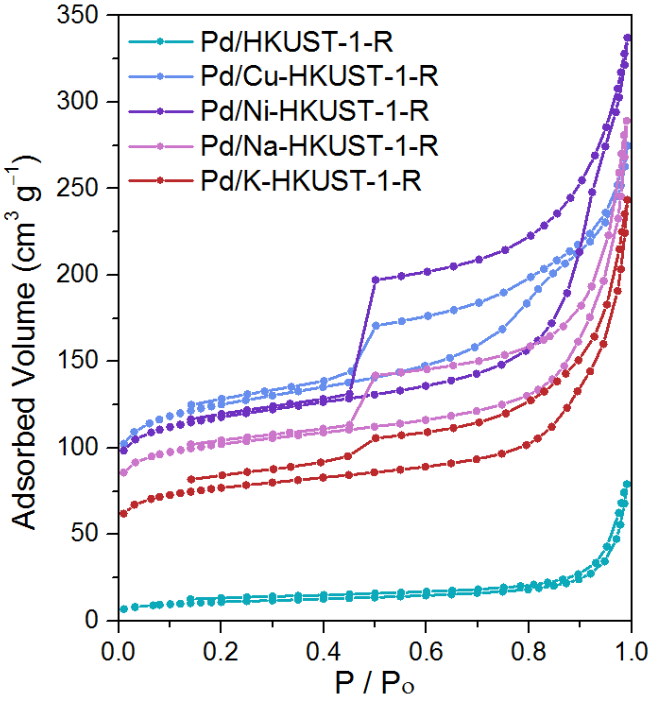

Supplementary Figure 5 N_2 adsorption-desorption isotherms of Pd/HKUST-1-R and its various composites after cation exchange.

From Supplementary Figure 5, it is clear that Pd/HKUST-1-R exhibits negligible porosity. This is due to the presence of CTA^+ counterions present in the pores of anionic HKUST-1-R support, which is in agreement to our FTIR measurement (Supplementary Figure 3). On the other hand, Pd/*M*-HKUST-1-R samples display hybrid type I/IV behaviours, which prove the coexistence of micropores and mesopores (Supplementary Figure 1). Similar to our previous work, the above samples exhibit H4-type hysteresis, which suggests the presence of slit-shaped mesopores[1].

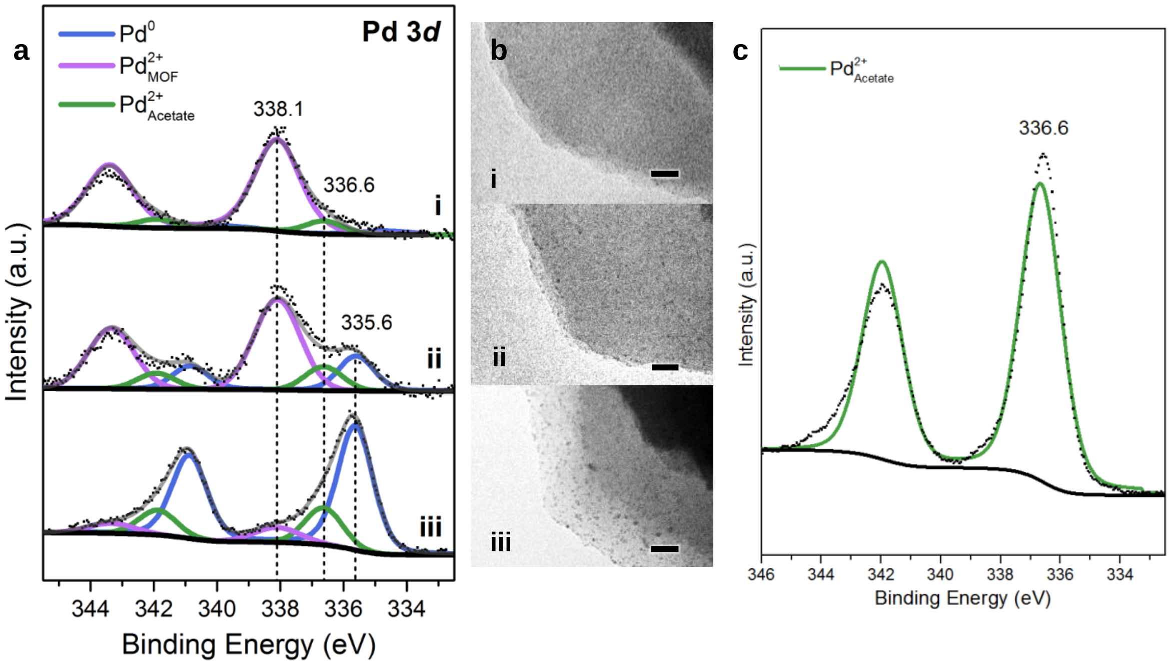

Supplementary Figure 6 Characterisation of Pd/HKUST-1-R prepared at different conditions and $Pd(OAc)_2$. **a,** High resolution XPS Pd 3*d* spectra of Pd/HKUST-1-R. **b,** TEM images of Pd/HKUST-1-R. Synthesis conditions after mixing HKUST-1-R with $Pd(OAc)_2$: (i) 5 min at room temperature, (ii) 12 h at room temperature, and (iii) 12 h at 60 °C. **c,** High resolution XPS Pd 3*d* spectra of $Pd(OAc)_2$. Scale bars: **b** 100 nm

With a reaction time of 5 min at room temperature, no Pd NP is observed on HKUST-1-R and its XPS peaks mainly correspond to the divalent Pd^{2+} species (Supplementary Figure 6ai). It is worth noting that a positive shift of the Pd^{2+} binding energy (BE) is observed when compared to the precursor palladium salt, i.e. $Pd(OAc)_2$ (Supplementary Figure 6c). This shows a strong interaction between the aromatic carboxylate groups on HKUST-1-R with Pd^{2+} after undergoing cation exchange with CTA^+. By increasing the reaction time to 12 h, appearance of Pd NPs and the coexistence of Pd^{2+} and Pd^0 XPS peaks are observed (Supplementary Figure 6aii). Upon increasing the reaction temperature to 60 °C, a denser population of Pd NPs is apparent while the XPS peaks mostly corresponds to the Pd^0 species (Supplementary Figure 6aiii).

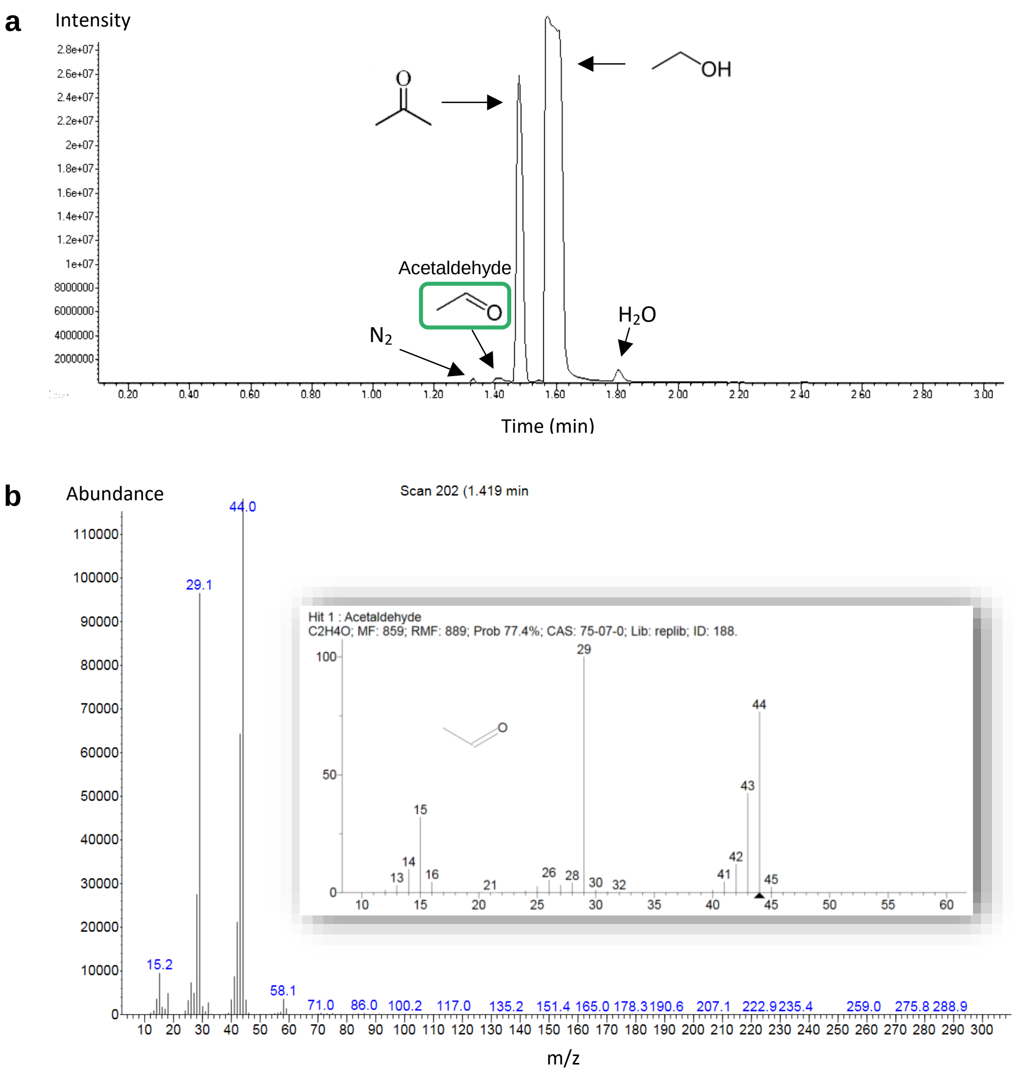

Supplementary Figure 7 Characterisation of products present during the preparation of Pd/HKUST-1-R. **a**, GC spectra of the supernatant. **b**, MS analysis of acetaldehyde present in the supernatant.

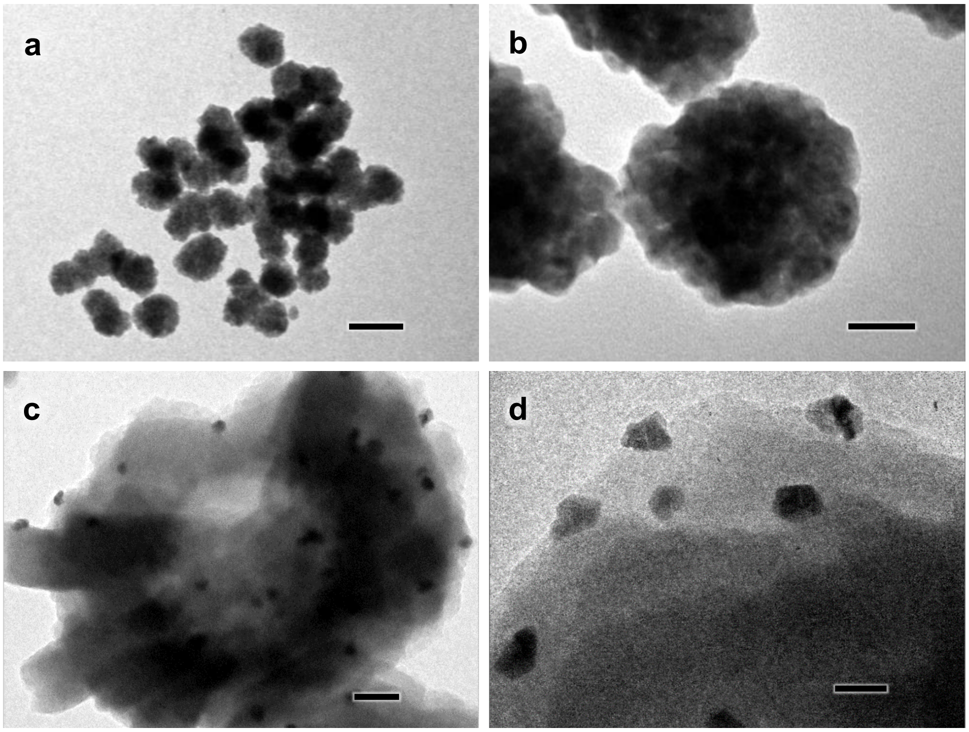

Supplementary Figure 8 TEM images at different magnifications of Pd NPs formed under different reaction conditions. **a**,**b**, Bare Pd NPs prepared at 60 °C without the use of HKUST-1-R under different magnification. **c**,**d**, Pd NPs reduced at 60 °C with the use of Cu-HKUST-1-R, i.e. MOF support without molecular-imprinted CTA^+, to form Pd-large/Cu-HKUST-1-R. Scale bars: **a** 80 nm, **b** 20 nm, **c** 100 nm, **d** 40 nm

Synthesis of bare Pd NPs (Supplementary Figure 8a,b). First, 3.20 mL of 5 mM $Pd(OAc)_2$ acetone solution was added to 90.0 mL of ethanol and this mixture was allowed to stir for 5 min. Subsequently, the solution was transferred into an oven controlled at 60 °C. After 12 h of reaction, the product was then collected, centrifuged and washed for 4 cycles using ethanol.

Synthesis of Pd-large/Cu-HKUST-1-R (Supplementary Figure 8c,d). First, 10.0 mL of Cu-HKUST-1-R ethanolic suspension was added to 80.0 mL of ethanol and this mixture was allowed to stir for 5 min. Subsequently, 3.20 mL of 5 mM $Pd(OAc)_2$ acetone solution was added dropwise over 1 min. The mixture was left to stir for another 5 min before transferring into an oven controlled at 60 °C. After 12 h of reaction, the product was then collected, centrifuged and washed for 4 cycles using ethanol and later suspended in 10.0 mL of ethanol.

In the former, large aggregates of Pd NPs of ~70 nm were formed (Supplementary Figure 8a,b). For the latter, Cu-HKUST-1-R contains no CTA^+ within the MOF structure, as a result, larger and less uniform Pd NPs were formed (Supplementary Figure 8c,d). These two control experiments reveal that Pd^{2+} can be reduced in ethanol at 60 °C, but size-control cannot be attained with or without MOF support alone. CTA^+ surfactant imprinted within HKUST-1-R is necessary to keep the particle size small and uniform.

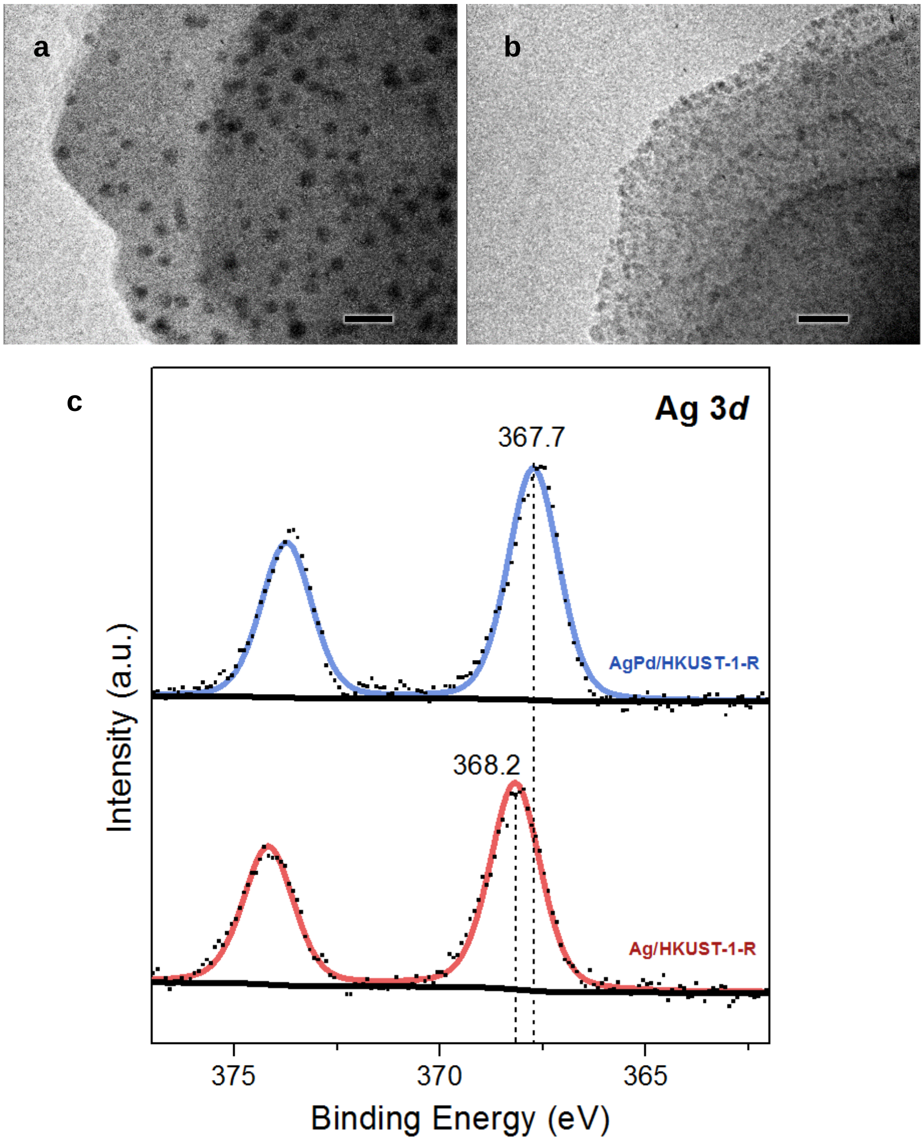

Supplementary Figure 9 Characterisation of Ag/HKUST-1-R and AgPd/HKUST-1-R. **a**, TEM image of Ag/HKUST-1-R. **b**, TEM image of AgPd/HKUST-1-R. **c**, High resolution XPS Ag 3*d* spectra of Ag/HKUST-1-R and AgPd/HKUST-1-R. Note that the decrease in the binding energy for AgPd/HKUST-1-R reveals the electron donating role of Pd, which indicates the formation of AgPd alloy NPs. Scale bars: **a** 30 nm, **b** 20 nm

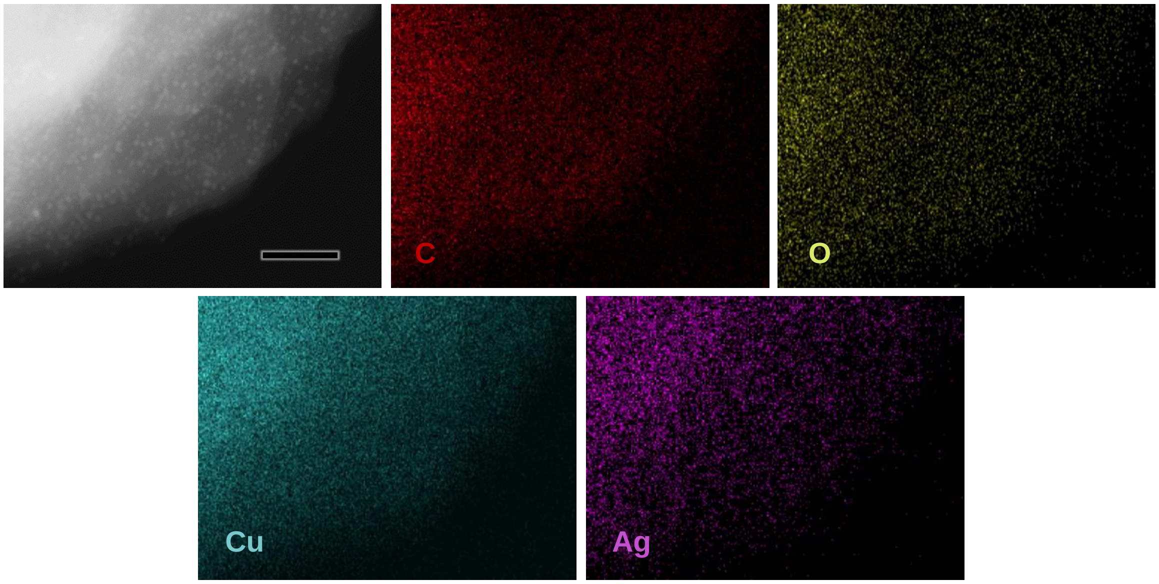

Supplementary Figure 10 Characterisation of Ag/HKUST-1-R. HAADF-STEM image of Ag/HKUST-1-R and its corresponding elemental mapping images. Scale bar: 150 nm

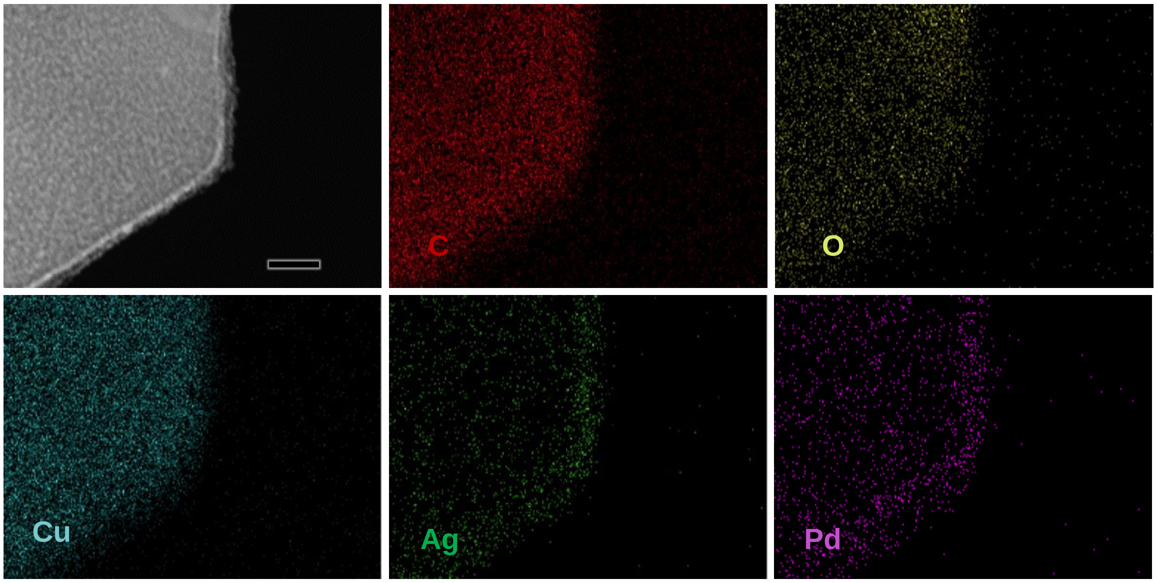

Supplementary Figure 11 Characterisation of AgPd/HKUST-1-R. HAADF-STEM image of AgPd/HKUST-1-R and its corresponding elemental mapping images. Scale bar: **5**0 nm

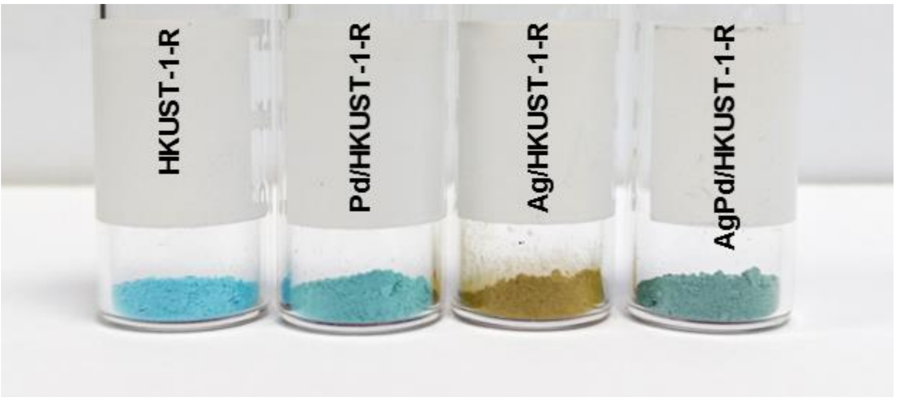

Supplementary Figure 12 Photographic image of the powdered samples of HKUST-1-R and its various noble metal composites. Photograph taken by Ying Chuan Tan.

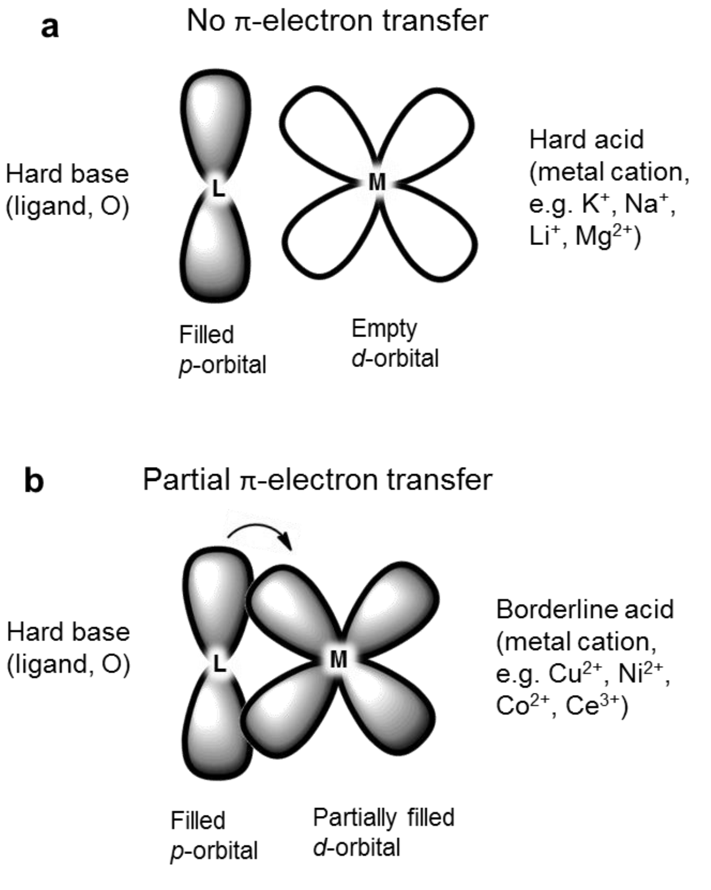

Supplementary Figure 13 Simplified illustration ligands-metal ions electronic interactions based on the hard/soft acid/base principle[2]. **a**, Interaction between hard base and hard acid. **b**, Interaction between hard base and borderline acid.

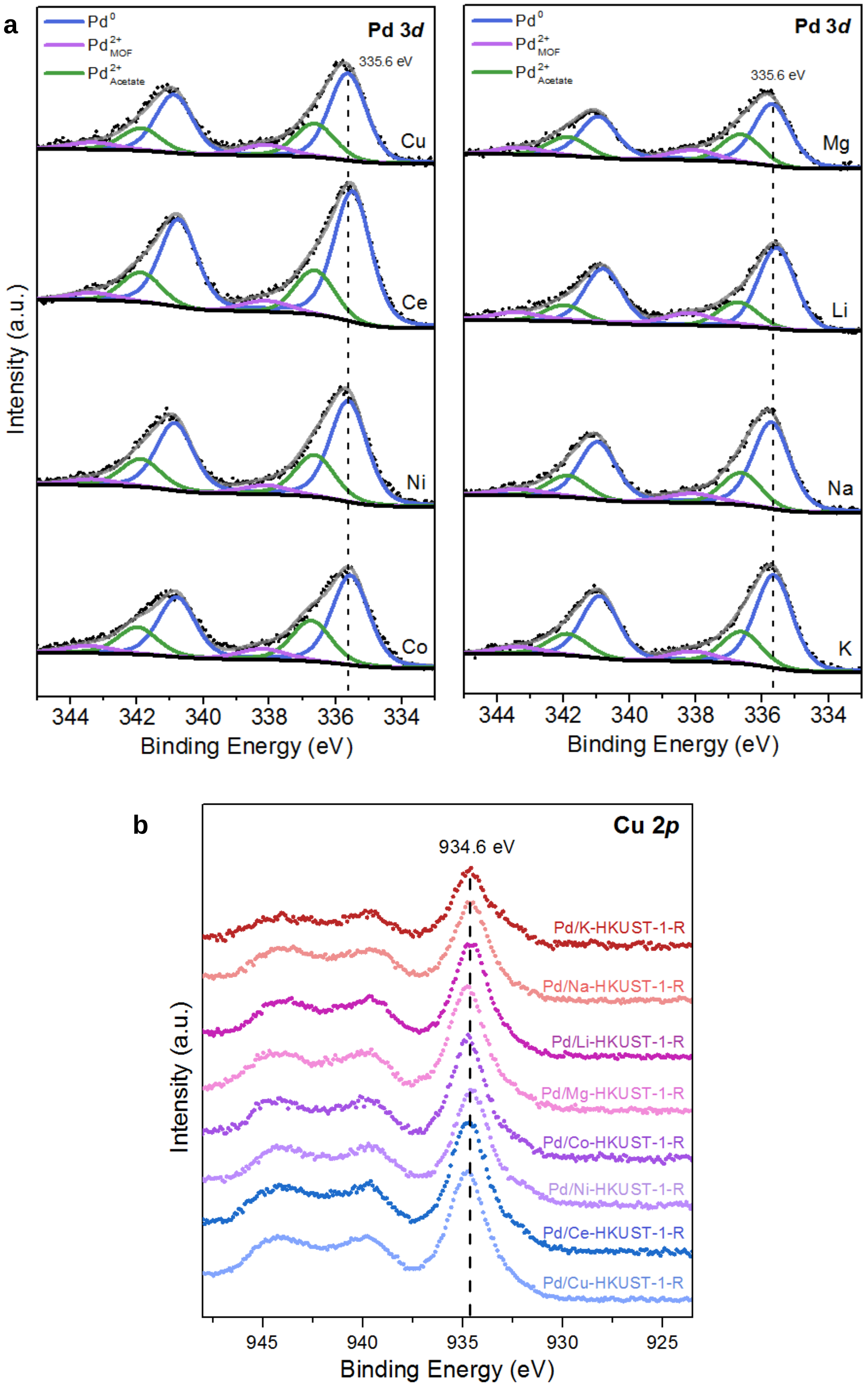

Supplementary Figure 14 Characterisation of Pd/*M*-HKUST-1-R. **a**, XPS Pd 3*d* spectra of the various cation-exchanged Pd/*M*-HKUST-1-R. **b**, XPS Cu 2*p* spectra of the various cation-exchanged Pd/*M*-HKUST-1-R.

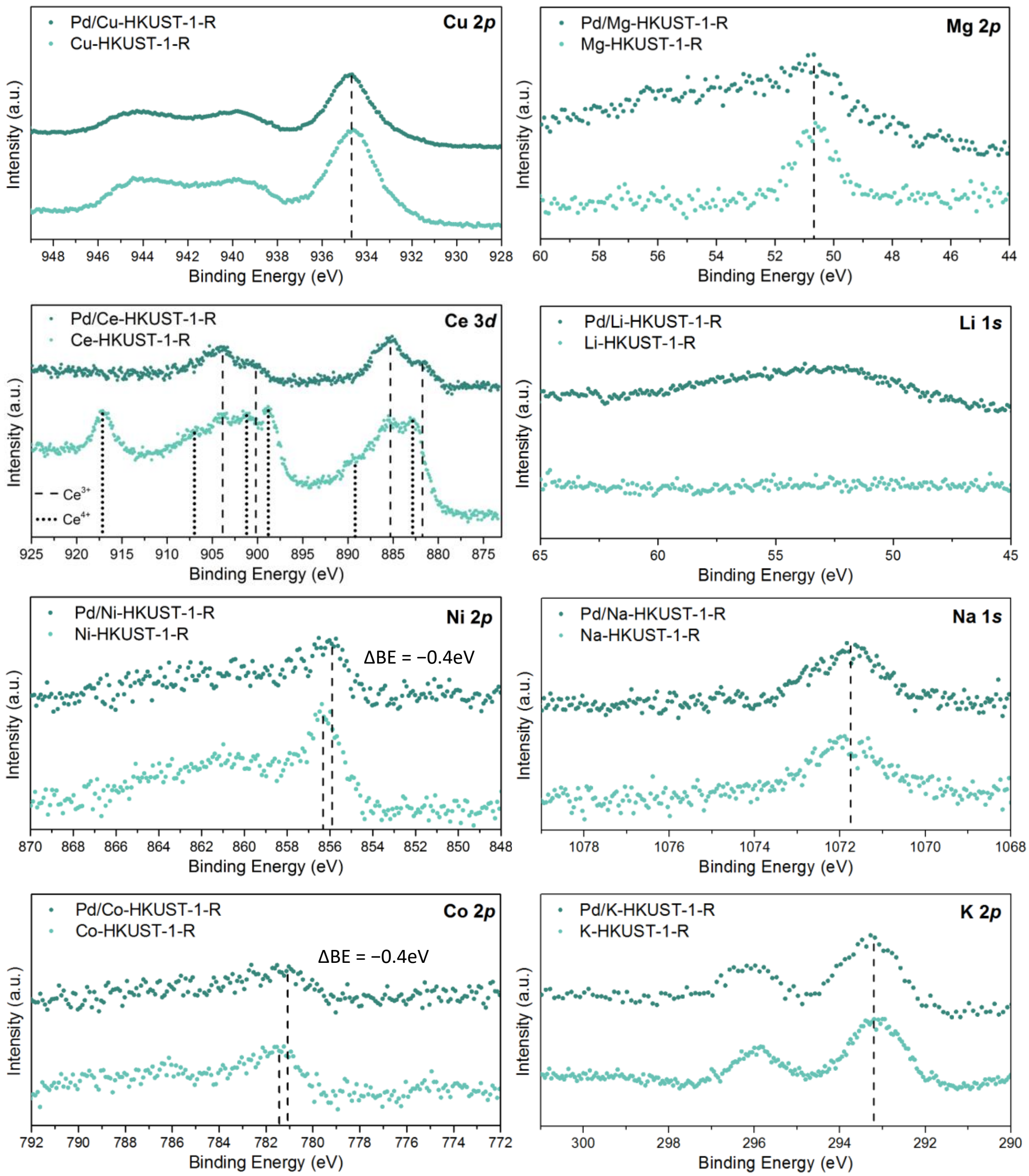

Supplementary Figure 15 Characterisation of Pd/*M*-HKUST-1-R and *M*-HKUST-1-R. Comparison of XPS spectra of the various cation-exchanged Pd/*M*-HKUST-1-R and *M*-HKUST-1-R.

Discussions for Supplementary Figures 14 and 15:

From Supplementary Figure 14a, XPS Pd 3*d* spectra of all Pd/*M*-HKUST-1-R samples show similar Pd^0 peaks at 335.6 eV, which indicate a lower electron density on the MOF-supported Pd NPs than unsupported care Pd NPs (Figure 4a). Therefore, the extent of electron transfer from the Pd NPs to the MOF support are comparable for all Pd/*M*-HKUST-1-R. In addition, XPS Cu 2*p* spectra of Pd/*M*-HKUST-1-R reveal no significant influence of the M^{n+} counterions on the electronic properties of Cu^{2+} species in the MOF support (Supplementary Figure 14b). Therefore, it is less likely that Cu^{2+} metal nodes of HKUST-1 framework participate in the electron transfer processes proposed in this work.

In Supplementary Figure 15, the XPS measurement signals of Pd/*M*-HKUST-1-R and *M*-HKUST-1-R are generally weak (except for Cu 2*p* signals) due to low metal ions loading on the surface of the samples. The XPS spectra of Li 1*s* of Pd/Li-HKUST-1-R and Li-HKUST-1-R did not show any noticeable peak. This is due to the low Li^+ population on the surfaces of samples and low sensitivity factors[3].

Clearly, XPS K 2*p*, Na 1*s* and Mg 2*p* spectra of the corresponding Pd/*M*-HKUST-1-R and *M*-HKUST-1-R samples indicate insignificant shift in binding energy due to the incorporation of Pd NPs in the MOF matrix. This is consistent with the hard/soft acid/base (HSAB) theory, which expects a low degree of electron transfer between the hard acid and hard base (oxygen from carboxylate groups). Therefore, the carboxylate groups are able to accumulate higher electron density that enable them to act as Lewis base (Figure 4c,d).

On the other hand, XPS Co 2*p* and Ni 2*p* spectra of the corresponding Pd/*M*-HKUST-1-R and *M*-HKUST-1-R samples show a negative shift in binding energy by 0.4 eV when Pd NPs are embedded within the MOF support. Interestingly, XPS Ce 3*d* spectra of Pd/Ce-HKUST-1-R and Ce-HKUST-1-R show a suppression in the formation of Ce^{4+} species in the presence of Pd NPs. These examples show that electron transfers from the pendant carboxylate groups to the Co^{2+}, Ni^{2+} and Ce^{3+} counterions are significant, thus reducing the electron density on the oxygen atoms. Hence, these observations are consistent with the HSAB principle, which explains the lack of basic property for these samples. However, XPS Cu 2*p* spectra of Pd/Cu-HKUST-1-R and Cu-HKUST-1-R show no significant change in binding energy. This is most likely due to the dilution of signals from Cu^{2+} counterions species among the signals from Cu^{2+} metal node species in the HKUST-1 framework.

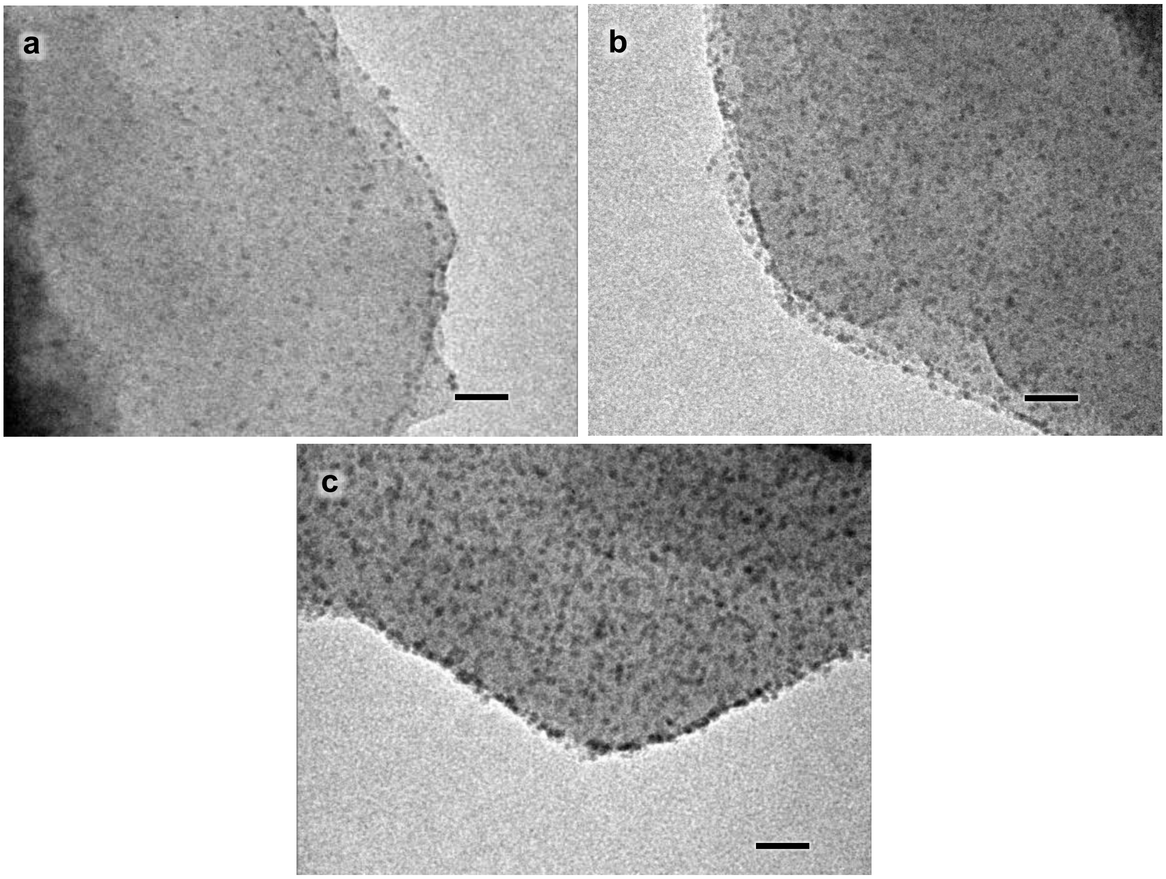

Supplementary Figure 16 TEM images of $Pd_{y\%}$/HKUST-1-R prepared from different amount of 5 mM $Pd(OAc)_2$ precursor (see Methods). **a**, $Pd_{0.5\%}$/HKUST-1-R was obtained using 1.60 mL of Pd^{2+} precursors. **b**, $Pd_{1.0\%}$/HKUST-1-R was obtained using 3.20 mL of Pd^{2+} precursors. **c**, $Pd_{2.6\%}$/HKUST-1-R was obtained using 8.00 mL of Pd^{2+} precursors. Note: Pd loadings achieved are determined by ICP-OES measurements. Scale bars: **a**–**c** 30 nm

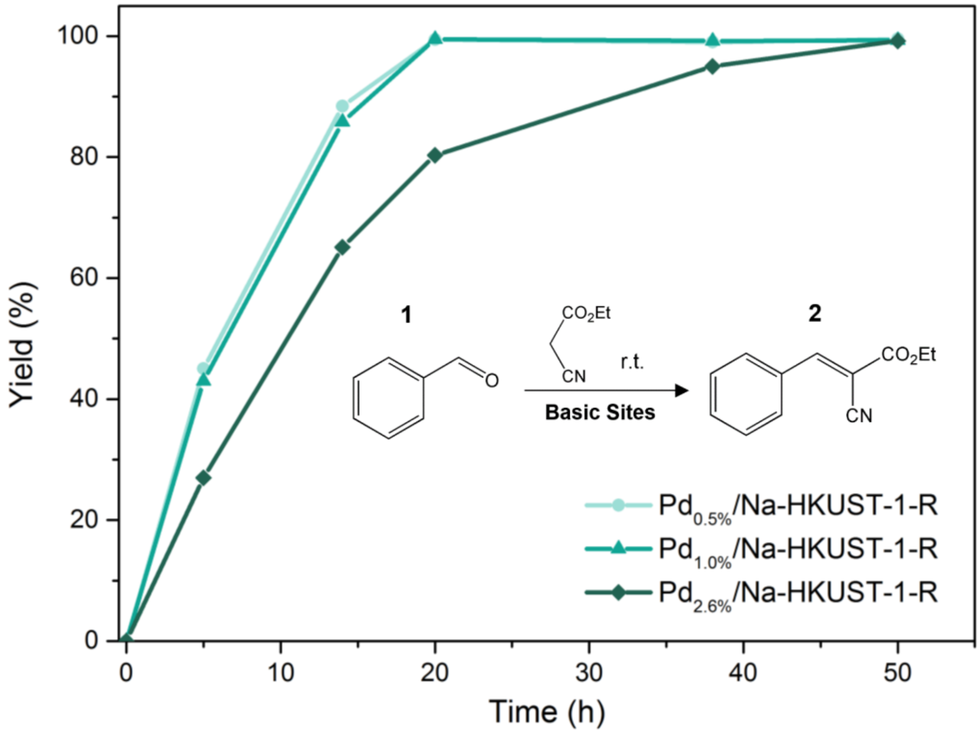

Supplementary Figure 17 Reaction profile of catalytic Knoevenagel condensation using $Pd_{y\%}$/Na-HKUST-1-R catalysts. Reaction conditions: benzaldehyde (1 mmol), ethyl cyanoacetate (1.5 mmol), ethanol (10 mL), $Pd_{y\%}$/Na-HKUST-1-R catalyst, room temperature and pressure. Yields are calculated with respect to **2**. For the above catalytic studies, the total amount of Pd in the catalysts added in each case was fixed at 0.5 mol% with respect to benzaldehyde, i.e. the amounts of $Pd_{1.0\%}$/Na-HKUST-1-R and $Pd_{2.6\%}$/Na-HKUST-1-R used in the latter two experiments were 50.0% and 19.2%, respectively, to that of $Pd_{0.5\%}$/Na-HKUST-1-R.

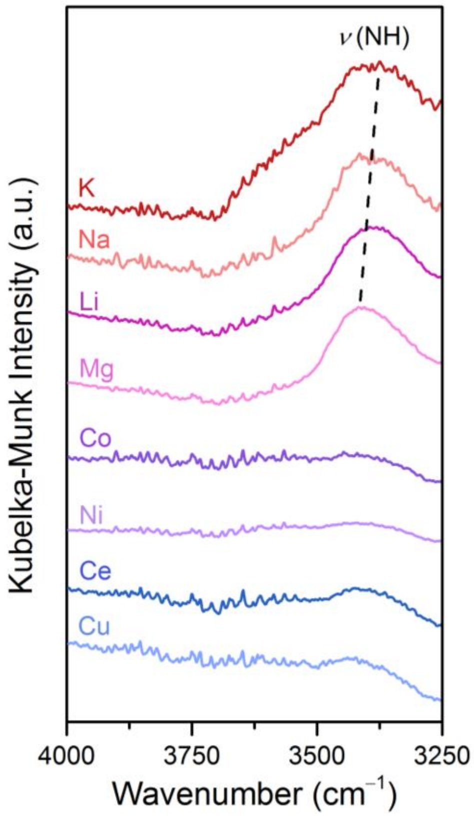

Supplementary Figure 18 Comparison of DRIFT spectra of pyrrole adsorbed on Pd/*M*-HKUST-1-R. DRIFTS measurements of all samples were conducted after exposing to pyrrole vapour and subsequent evacuation under nitrogen flow at 60 °C.

From the DRIFT spectra of Pd/*M*-HKUST-1-R for *M*: Mg, Li, Na and K, presented in Supplementary Figure 18, the apparent broad bands between 3410 and 3350 cm^{-1} can be assigned to the stretching vibration of the NH group of pyrrole molecule[4]. More specifically, the band at 3410 cm^{-1} is assigned to the vibration of the NH group interacting with the ring of another pyrrole molecule to form a NH–π complex[5]. On the other hand, the interaction between pyrrole molecule and H-bond acceptor (*i.e.* basic site on Pd/*M*-HKUST-1-R) would result in a bathochromic shift of the NH band and the extent of this shift is correlated to the basic strength of the adsorption site[4,5]. It is likely that the broad bands observed in the DRIFT spectra are a result of overlapping absorption bands composed of these two distinct types of pyrrole interaction. Nevertheless, the bathochromic shifts of the NH bands are still observable, which increase in the order: Pd/Mg-HKUST-1-R < Pd/Li-HKUST-1-R < Pd/Na-HKUST-1-R ≈ Pd/K-HKUST-1-R. This trend can be positively correlated to the proton affinity and the ease of donating electron pair to the proton (*i.e.* Lewis basic strength) of their corresponding pendant carboxylate sites. For Pd/*M*-HKUST-1-R for *M*: Cu, Ce, Ni and Co, it is clear that their spectra show negligible absorption band of the NH group, which indicate a lack of basic sites strong enough to interact with the acidic pyrrole molecule. The results obtained from the DRIFTS measurements is consistent with the catalytic performance of the Pd/*M*-HKUST-1-R summarized in Table 1 and further support the role that M^{n+} counterions play in influencing the properties of pendant carboxylate sites as presented in Figure 4.

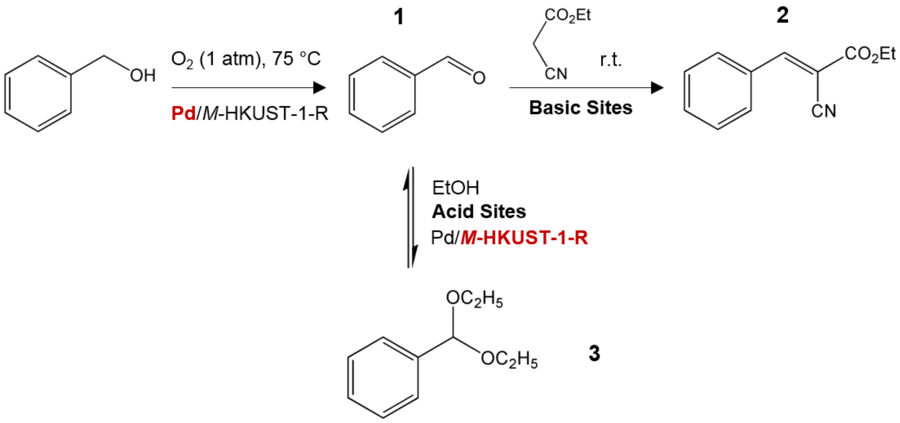

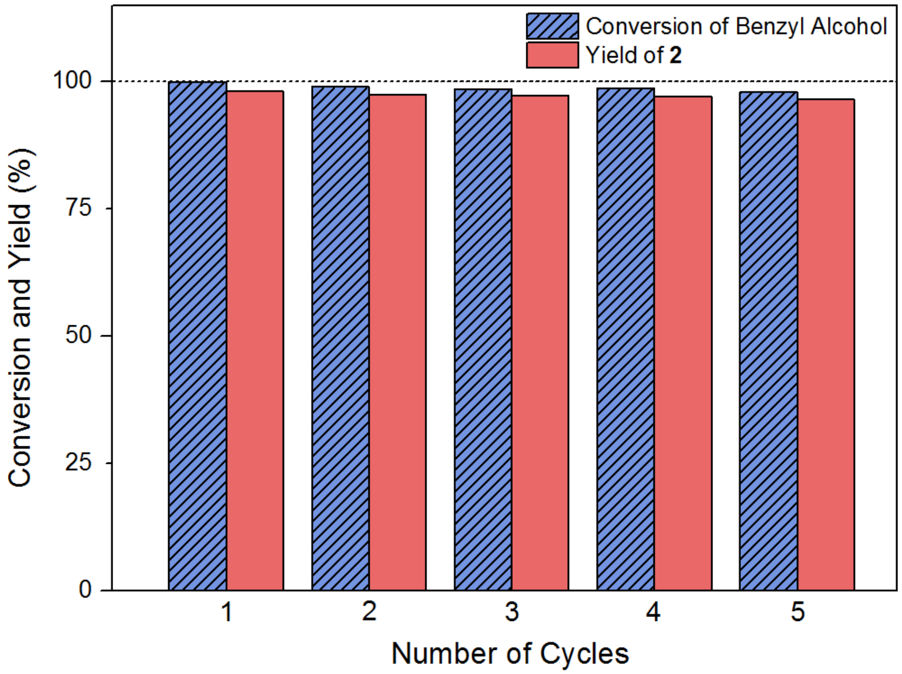

Supplementary Figure 19 Catalytic stability of Pd/Na-HKUST-1-R. Oxidation-Knoevenagel condensation reactions were carried out for 5 cycles, and only a small drop of catalytic performance was observed (could be resulted from the loss of catalysts during the washing step in between cycles). Functional catalytic components of Pd/*M*-HKUST-1-R in the above three reactions are indicated in red.

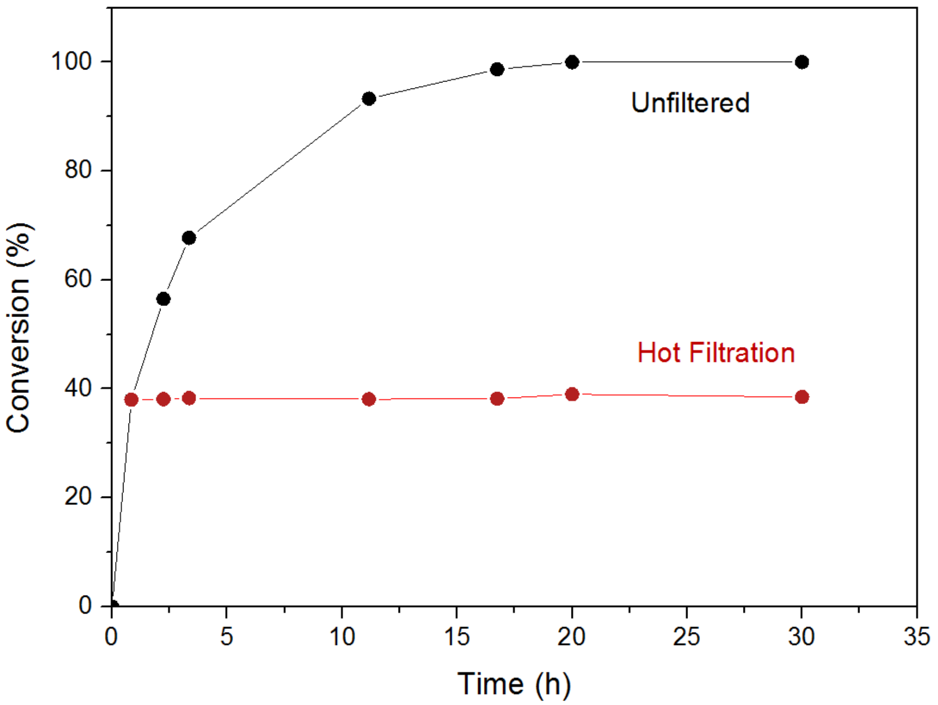

Supplementary Figure 20 Catalyst leaching test. Conversion of benzyl alcohol oxidation over time with and without filtering off Pd/Na-HKUST-1-R catalysts. The oxidation reaction was halted when the catalysts were removed, which indicated the reaction is catalysed heterogeneously and Pd content was not leached out.

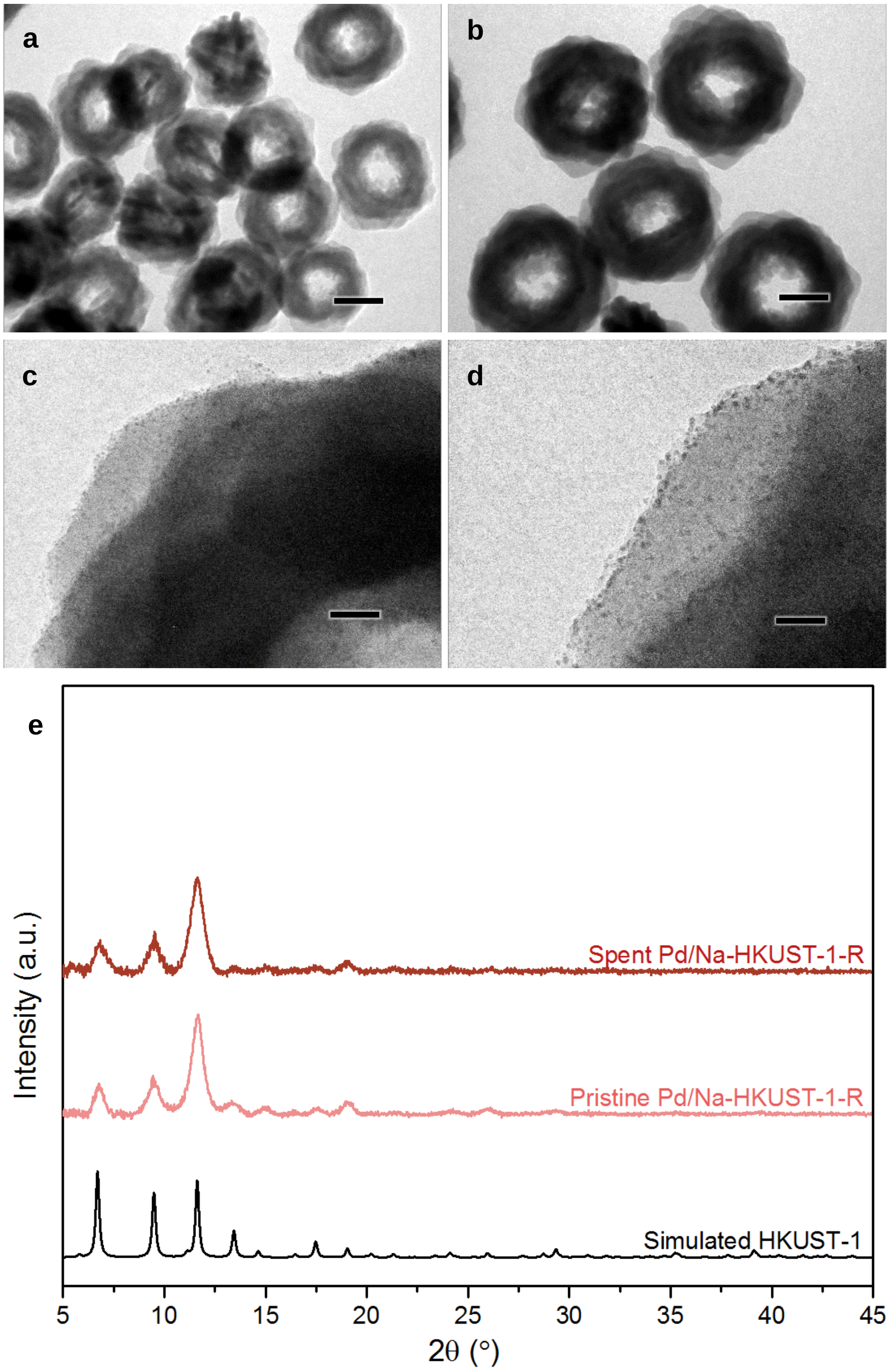

Supplementary Figure 21 Characterisation of Pd/Na-HKUST-1-R after undergoing stability tests for oxidation-Knoevenagel condensation reactions (5 cycles). **a**–**d**, TEM image of spent Pd/Na-HKUST-1-R at different magnification. Clearly, the overall morphology of the MOF support was retained and the Pd NPs did not agglomerate after the stability tests. **e**, PXRD measurements of Pd/Na-HKUST-1-R before and after undergoing stability tests. The crystallinity of the MOF support showed no obvious change after the cycled reactions. Scale bars: **a** 600 nm, **b** 400 nm, **c** 80 nm, **d** 50 nm

Supplementary Tables

Supplementary Table 1 Summary of metal composition in Pd/*M*-HKUST-1-R measured from ICP-OES

Sample	*M* (mol %)	Cu (mol %)	Pd (mol %)
Pd/Cu-HKUST-1-R	-	97.2	2.8
Pd/Ce-HKUST-1-R	8.7	88.7	2.6
Pd/Ni-HKUST-1-R	7.5	90.0	2.5
Pd/Co-HKUST-1-R	6.8	90.6	2.6
Pd/Mg-HKUST-1-R	8.2	89.0	2.8
Pd/Li-HKUST-1-R	9.6	87.6	2.8
Pd/Na-HKUST-1-R	13.9	83.4	2.7
Pd/K-HKUST-1-R	11.1	86.4	2.5

Supplementary Table 2 Textural Properties of Pd/HKUST-1-R and its various composites after cation exchange

Sample	V_{total} ($cm^3\ g^{-1}$)	S_{BET} ($m^2\ g^{-1}$)
Pd/HKUST-1-R	0.110	39.6
Pd/Cu-HKUST-1-R	0.288	473.0
Pd/Ni-HKUST-1-R	0.438	448.2
Pd/Na-HKUST-1-R	0.386	390.6
Pd/K-HKUST-1-R	0.286	291.2

Supplementary Table 3 Summary of catalytic Knoevenagel condensation[a]

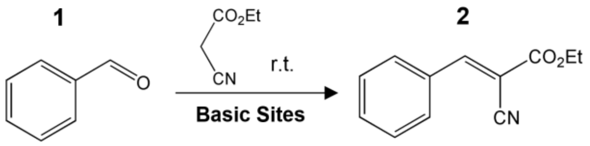

Catalyst	Conversion (%)	Selectivity [b] (%)
Pd/Cu-HKUST-1-R	> 99	0
Pd/Na-HKUST-1-R	> 99	> 99
Cu-HKUST-1-R	> 99	0
Na-HKUST-1-R	56	2

[a] Reaction conditions: benzaldehyde (1 mmol), ethyl cyanoacetate (1.5 mmol), ethanol (10 mL), Pd/*M*-HKUST-1-R (0.5 mol% Pd), room temperature and pressure, 24 h. [b] With respect to **2**. For Pd/Cu-HKUST-1-R, Cu-HKUST-1-R and Na-HKUST-1-R, the major by-product of this catalytic studies is benzaldehyde diethyl acetal due to the presence of Lewis acid sites that catalyse the acetalisation between benzaldehyde and ethanol.

Supplementary Table 4 Pd/*M*-HKUST-1-R catalysed benzyl alcohol oxidation reaction[a]

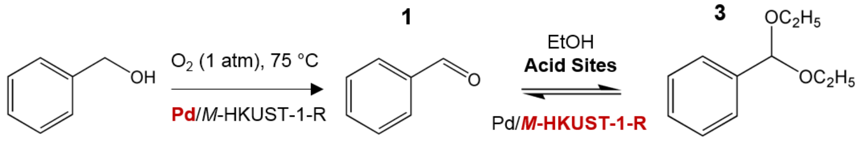

Catalyst	Conversion (%)	Yield (%)	
		**1**	**3**
Pd/Cu-HKUST-1-R	> 99	44.3	55.7
Pd/Ce-HKUST-1-R	> 99	45.2	54.8
Pd/Ni-HKUST-1-R	> 99	48.5	51.5
Pd/Co-HKUST-1-R	> 99	50.9	49.1
Pd/Mg-HKUST-1-R	> 99	97.0	3.0
Pd/Li-HKUST-1-R	> 99	98.7	1.3
Pd/Na-HKUST-1-R	> 99	99.1	0.9
Pd/K-HKUST-1-R	> 99	100.0	0.0

[a] Reaction conditions: benzyl alcohol (1 mmol), ethanol (10 mL), Pd/*M*-HKUST-1-R (0.5 mol% Pd), 75 °C, in flowing O_2 gas (1 atm), 20 h. Functional catalytic components of Pd/*M*-HKUST-1-R in the above two reactions are indicated in red.

Supplementary References

1 Tan, Y. C. & Zeng, H. C. Defect creation in HKUST-1 via molecular imprinting: attaining anionic framework property and mesoporosity for cation exchange applications. *Adv. Funct. Mater.* **27**, 1703765 (2017).

2 Glusker, J. P. Structural aspects of metal liganding to functional groups in proteins. *Adv. Protein Chem.* **42**, 1-76 (1991).

3 Ward, R. & Wood, B. A comparison of experimental and theoretically derived sensitivity factors for XPS. *Surf. Interface Anal.* **18**, 679-684 (1992).

4 Murphy, D., Massiani, P., Franck, R. & Barthomeuf, D. Basic site heterogeneity and location in alkali cation exchanged EMT zeolite. An IR study using adsorbed pyrrole. *J. Phys. Chem.* **100**, 6731-6738 (1996).

5 Valvekens, P., Vandichel, M., Waroquier, M., Van Speybroeck, V. & De Vos, D. Metal-dioxidoterephthalate MOFs of the MOF-74 type: microporous basic catalysts with well-defined active sites. *J. Catal.* **317**, 1-10 (2014).
